# Safety and tolerability of moxidectin and ivermectin combination treatments for lymphatic filariasis in Côte d’Ivoire: A randomized controlled superiority study

**DOI:** 10.1371/journal.pntd.0011633

**Published:** 2023-09-18

**Authors:** Catherine M. Bjerum, Benjamin G. Koudou, Allassane F. Ouattara, Daphne Lew, Charles W. Goss, Pascal T. Gabo, Christopher L. King, Peter U. Fischer, Gary J. Weil, Philip J. Budge

**Affiliations:** 1 Center for Global Health and Diseases, Case Western Reserve University School of Medicine, Cleveland, Ohio, United States of America; 2 Université Nangui Abrogoua, Abidjan, Côte d’Ivoire; 3 Centre Suisse de Recherche Scientifique en Côte d’Ivoire, Abidjan, Côte d’Ivoire; 4 Division of Biostatistics, Washington University School of Medicine, St. Louis, Missouri, United States of America; 5 Centre Hospitalier Régional d’Agboville, Agboville, Côte d’Ivoire; 6 Veterans Affairs Research Service, Cleveland Veterans Affairs Medical Center, Cleveland, Ohio, United States of America; 7 Infectious Diseases Division, Department of Medicine, Washington University School of Medicine, St. Louis, Missouri, United States of America; Uniformed Services University: Uniformed Services University of the Health Sciences, UNITED STATES

## Abstract

**Background:**

Moxidectin is a macrocyclic lactone registered for the treatment of human onchocerciasis. The drug has a good safety profile, large volume of distribution and a long elimination half-life. This paper reports tolerability data from the first use of moxidectin in persons with *Wuchereria bancrofti* infection.

**Methods:**

In this randomized, open-label, masked-observer superiority trial, adults with *Wuchereria bancrofti* microfilaremia in Côte d’Ivoire were randomized to 1 of 4 treatment arms: ivermectin + albendazole (IA), moxidectin + albendazole (MoxA), ivermectin + diethylcarbamazine (DEC) + albendazole (IDA), or moxidectin + DEC + albendazole (MoxDA). As part of a larger efficacy trial, all participants were closely monitored for 7 days after treatment.

**Results:**

One hundred sixty-four individuals were treated, and monitored for treatment emergent adverse events (TEAE). Eighty-seven participants (53%) experienced one or more mild (grade 1) or moderate (grade 2) TEAE. Four participants had transient Grade 3 hematuria after treatment (3 after IDA and 1 after IA). There were no serious adverse events. There were no significant differences in frequency or types of TEAE between treatment groups (IA = 22/41 (53%), MoxA = 24/40 (60%), IDA = 18/41 (44%), MoxDA = 15/42 (36%), p = 0.530). Fifty-nine participants (36%) had multiple TEAE, and 8.5% had a one or more grade 2 (moderate) TEAE. Grade 2 TEAE were more frequent after triple drug treatments (IDA, 14.6%; MoxDA, 9.5%) than after two-drug treatments (IA, 7.3%; MoxA, 2.5%). There was no difference in TEAEs based on baseline Mf counts (OR 0.69 (0.33, 1.43), p-value 0.319).

**Conclusion:**

All treatment regimens were well tolerated. We observed no difference in safety parameters between regimens that contained ivermectin or moxidectin.

**Trial registration:**

Clinicaltrials.gov, NCT04410406.

## Author summary

Lymphatic Filariasis (LF) is a mosquito-borne parasitic infection caused predominantly by *Wuchereria bancrofti*. Infection can lead to significant lymphatic dysfunction, including hydroceles and lymphedema, which can progress to elephantiasis. The World Health Organization’s Global Programme to Eliminate LF (GPELF) recommends mass drug administration (MDA) in endemic populations to eliminate the disease. GPELF recommendations for MDA include ivermectin (IVM) plus albendazole (IA) in sub-Saharan Africa where onchocerciasis is present. In 2018 the US Food and Drug Administration approved use of moxidectin for treatment of onchocerciasis. Moxidectin is a macrocytic lactone, similar to IVM, but more lipophilic, with a larger volume of distribution and longer half-life. Onchocerciasis studies found moxidectin to be superior to IVM for clearance of microfiladermia in people with onchocerciasis, with a treatment emergent adverse event (TEAE) profile similar to that of ivermectin. Moxidectin has not yet been studied, alone or in combination with other antihelminthic drugs. This is a safety evaluation of the first trial of moxidectin combination therapy for LF, which shows that moxidectin combination regimens are well tolerated in *Wuchereria bancrofti-*infected patients, with a TEAE profile comparable to standard ivermectin containing regimens.

## Introduction

Lymphatic Filariasis (LF) is a mosquito-borne parasitic nematode infection caused by *Wuchereria bancrofti*, *Brugia*. *malayi* or *B*. *timori*. Infection can lead to significant lymphatic dysfunction, hydroceles, and limb lymphedema, which can progress to elephantiasis. The World Health Organization’s (WHO) Global Programme to Eliminate LF (GPELF) recommends mass drug administration (MDA) in endemic populations to eliminate the disease. GPELF is the largest MDA-based infectious disease intervention program attempted to date with over 9 billion doses of medications distributed between 2000 and 2021. Global efforts to eliminate LF transmission have reduced the population at risk for LF, but MDA is still recommended for nearly 885 million people in 45 countries [1]. Previous GPELF recommendations for MDA include ivermectin plus albendazole (IA) in sub-Saharan Africa where onchocerciasis is present, albendazole twice yearly in areas co-endemic for LF and *Loa loa* and diethylcarbamazine (DEC) plus albendazole (DA) in other endemic areas. However, after clinical trials in Papua New Guinea and Côte d’Ivoire found the combination of ivermectin plus DEC plus albendazole (IDA) to be superior to 2-drug combinations [2–4], and subsequent large, multinational safety trials in over 26,000 participants showed no increase in treatment emergent adverse events (TEAEs) with IDA compared to DA [5], WHO endorsed IDA for MDA in certain settings without onchocerciasis or loiasis [6]. However, IDA cannot be used in sub-Saharan Africa because DEC can precipitate serious adverse ocular events in persons with onchocerciasis. There remains a need for safe and more efficacious treatment that can be used for MDA in LF elimination programs in onchocerciasis co-endemic regions.

In 2018 the US Food and Drug Administration approved use of moxidectin for treatment of onchocerciasis [7]. Moxidectin is a macrocytic lactone, similar to IVM, but more lipophilic, with a larger volume of distribution and longer half-life. Onchocerciasis studies conducted in Liberia, Democratic Republic of Congo and Ghana found moxidectin to be superior to IVM for clearance of microfiladermia in people with onchocerciasis, with a TEAE profile similar to that of ivermectin (7). Moxidectin has not yet been studied, alone or in combination with other antihelminthic drugs, for safety or efficacy against LF. Here we report the study design and safety evaluation of the first trial of moxidectin combination therapy for LF.

## Methods

### Ethics statement

The study protocol and related documents were approved by the institutional review boards in St. Louis (Washington University, IRB#202005076), Cleveland, USA (Case Western Reserve University, IRB#STUDY20200714) and in Côte d’Ivoire (CNESVS #011-20/MSHP/CNESVS-km). This trial is registered at Clinicaltrials.gov (NCT04410406).

### Study design

**Fig 1:**
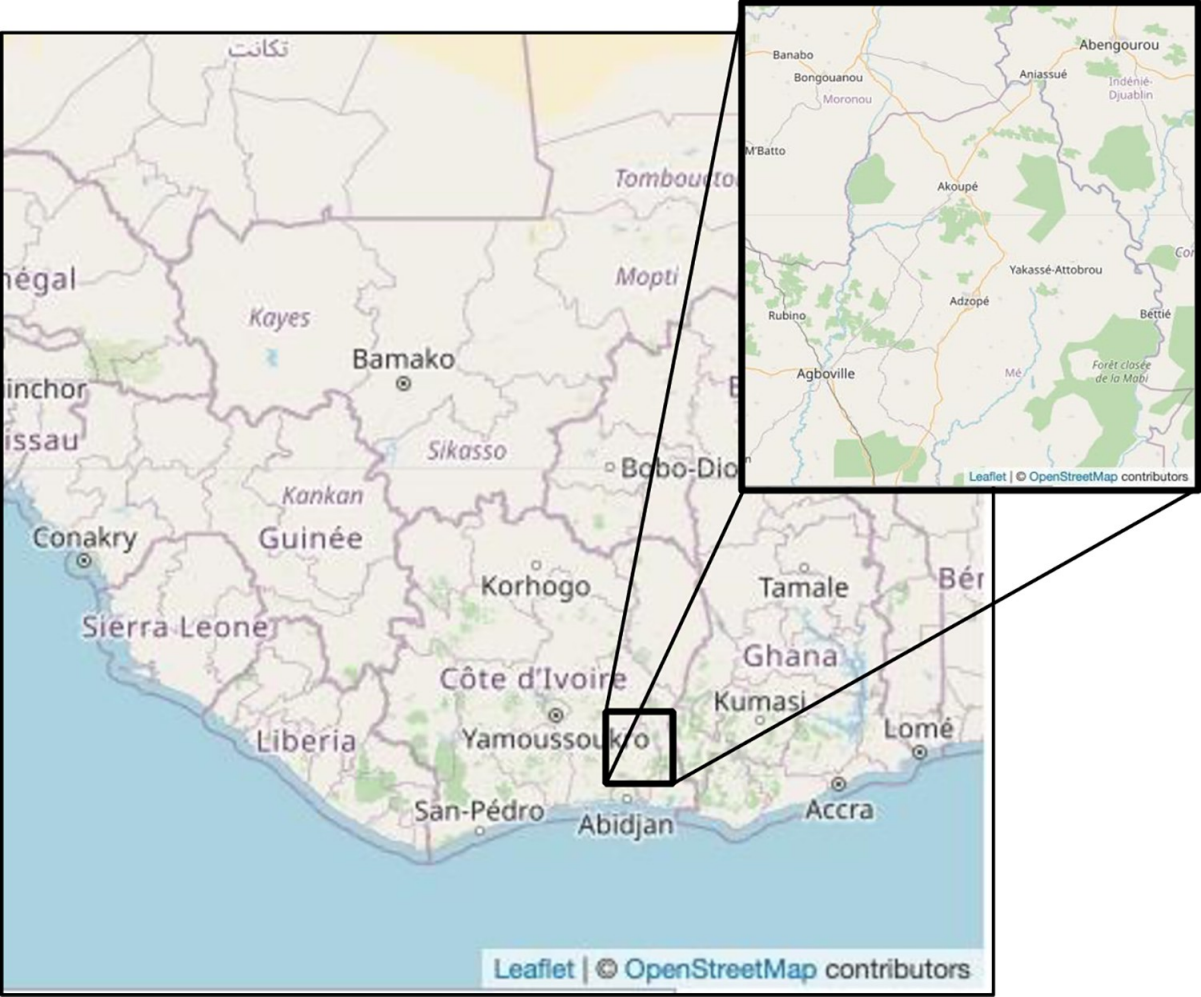
Map of study location. Base map: https://www.openstreetmap.org/#map=5/8.798/-3.010.

This single-site, Phase III, randomized, open-label, masked-observer superiority trial includes four treatment arms: ivermectin 200μg/kg + albendazole 400mg (IA), moxidectin 8mg + albendazole 400mg (MoxA), ivermectin 200μg/kg + DEC 6mg/kg + albendazole 400mg (IDA), and moxidectin 8mg + DEC 6mg/kg + albendazole 400mg (MoxDA). Primary study endpoints are the proportion of participants having complete clearance of microfilaremia at 12 months (for the IA vs. MoxA comparison) and at 24 months (for IDA vs. MoxDA comparison). Secondary endpoints include the frequency and severity of TEAEs during the first 7 days after treatment. Participants were recruited from Agboville, Akoupé, Abengourou, and Bongounou Health Districts, in eastern Côte d’Ivoire (Fig 1). These areas are endemic for LF and onchocerciasis, but non-endemic for loiasis. Participants eligible for inclusion in the study were healthy non-pregnant, non-breast feeding, *Wuchereria bancrofti*-infected adults predicted to have at least 40 *W*. *bancrofti* microfilariae (Mf) per mL of blood based on the results of a pre-screening 60 μL nocturnal blood smear. This Mf cutoff was chosen to ensure the primary efficacy endpoint of the study, which is complete clearance of microfilaremia, would be meaningful and not met by random variation due to low starting Mf counts. Individuals who assessed adverse events were blinded to treatment arm.

### Parasitology testing

Prescreening for *W*. *bancrofti* infection was performed by the Programme National de Lutte Contre la Schistosomiases, les Géohelminths et la Filariose Lymphatique (PNL-SGF) within the Ivorian Ministry of Health using a rapid test for circulating filarial antigenemia (Filariasis Test Strip or FTS). Those with a positive FTS result had blood collected between 21:30 and 23:30 for Mf testing by a calibrated, three-line 60 μL thick blood smear. At the time of enrollment, and at all subsequent study time-points, Mf testing was done via membrane filtration of one mL of anticoagulated venous blood. Two microscopists independently read Giemsa-stained filters to assess Mf counts.

At the time of enrollment, all participants were screened for onchocerciasis with skin biopsies (“skin snips”) over each iliac crest. Participants with onchocerciasis, defined as one or more *Onchocerca volvulus* Mf present in either skin snip, were excluded from the study.

### Participants and enrollment

The study was conducted in two parts. The first part involved 72 patients enrolled between August 20, 2020 and September 22, 2020. It included inpatient treatment with intensive safety monitoring at the Centre de Recherche de Filariose Lymphatique d’Agboville, located at Centre Hôspitalier Regional d’Agboville, Côte d’Ivoire for the first 72 hours after treatment and at day 7, with passive outpatient safety monitoring in their village on days 4–6. After observing no concerning safety signals from this cohort, the data safety monitoring board approved the prespecified plan to treat the remaining patients in their villages with daily outpatient TEAE monitoring. Subsequent enrollments (part II) took place between December 1, 2020 and July 30, 2021.

Eligible participants included healthy adults aged 18–70 years old, with no recent acute illness, and no antifilarial treatment within the past year. Contraception prior to, and for 1 month following, treatment was required for all women of childbearing age. Pregnant or breastfeeding women were excluded. Exclusion criteria included: history of chronic kidney or liver disease; creatinine, serum alanine transaminase or aspartate transaminase level >2 times the upper limits of normal (measured by a portable biochemistry analyzer); blood hemoglobin <7 gm/dL; history of prior allergic reaction to study medications; coinfection with *O*. *volvulus*, or self-reported use of any medications that could interfere with test drug metabolism within one week of study onset (listed in supporting information**)**. Participants without exclusionary conditions were enrolled, randomized, and treated before results of their baseline nocturnal Mf counts were available; in some cases this resulted in enrollment of participants with fewer than 40 Mf/mL, the pre-specified threshold for inclusion in the efficacy analysis. However, all participants receiving study drugs, regardless of baseline Mf counts, were included in this safety analysis. This change to inclusion criteria was approved by all IRBs and ethic committees. All participants signed a written informed consent prior to screening and enrollment in the study. The full study protocol is available as Supplemental Information 1.

### Treatment

Participants were randomized to one of the following oral, single-dose combination therapies: ivermectin 200 μg/kg plus albendazole 400 mg (IA), given annually (standard of care for LF MDA in Cote d’Ivoire), moxidectin 8 mg plus albendazole 400 mg (MoxA), ivermectin 200 μg/kg plus DEC 6 mg/kg plus albendazole 400 mg (IDA), or moxidectin 8 mg plus DEC 6 mg/kg plus albendazole 400 mg (MoxDA). Study medications were prepared and administered by an unblinded pharmacist, according to a pre-designated block randomization list. No other personnel had access to the randomization or to which arm participants were randomized. Participants and all individuals performing laboratory tests, assessing TEAE, performing physical or ultrasound exams were blinded to study arm.

### Blood collection and assessment of adverse events

For part I, groups of 10 same sex participants were brought to the research center the night prior to treatment for screening, which included baseline laboratory tests, physical exams and scrotal ultrasound examinations for men. The following morning, starting at 7 a.m., and within about 30 minutes after breakfast, participants were treated with a single co-administered dose of the study medications according to the randomization scheme. All study medications were administered as directly observed therapy and participants were observed for 30 minutes after administration. Biochemistry and urine tests were repeated at 24 and 48 hours and 7 days post-treatment. There was 100% compliance with all blood draws for the pharmacokinetic samples as well as biochemistry laboratory assessments. TEAE is defined as an undesirable event that emerges during treatment, having been absent pre-treatment, or worsens relative to the pre-treatment state. After treatment, participants were monitored for TEAEs every 6 hours for the first 48 hours, then every 12 hours until 72 hours, and again at day 7 post-treatment. Upon returning to their home village after the first 72 hours, passive surveillance for potential TEAEs was conducted by trained community health workers in the participants’ home villages on days 4–6 with active follow up on day 7 by study personnel. New or worsening symptoms, changes in vital signs, or new abnormal findings on laboratory tests or physical examination were considered to be TEAEs and were scored using a modified version of the National Cancer Institute Common Terminology Criteria for Adverse Events, v4.0. We evaluated both objective and subjective TEAEs. Subjective TEAEs were symptoms felt and reported by the participants, while objective TEAEs were measured laboratory values, vital signs or physical exam findings.

After a pre-specified review of safety data from Part I (inpatient monitoring), the DSMB recommended continuation to part II (outpatient treatment and monitoring). Participants in part II were enrolled and treated in their village of residence, applying the same screening and eligibility criteria as part I. Active follow up of all participants was performed in their village at 24 and 48 hours after treatment. Biochemistry and urine tests were repeated at 48 hours after treatment. Passive surveillance was performed by trained village health workers through day 7. Any grade 2 (moderate) or higher TEAE was followed until resolution or stabilization.

### Randomization and statistical analysis

Sample size was determined based on anticipated efficacy outcomes. A prior clinical trial in Cote d’Ivoire found that IA achieved complete clearance of Mf in 26% of participants at 12 months^3^. A sample size of 44 per group would have 80% power to show a statistically significant difference between MoxA and IA, if MoxA achieved clearance in 55% of participants at 12 months (compared to 26% for IA). This same trial found that IDA achieved prolonged clearance of Mf in about 50% of participants at 24 months. This study is powered to test the hypothesis that 80% clearance will be seen after MoxDA compared to 50% after IDA at 24 months. A sample size of 39 per group would be required to have 80% power to detect such a difference in proportions with 2-sided type 1 error rate of 0.05. Assuming a loss to follow up rate of up to 22%, we would need to enroll 50 participants per arm (200 total) to be able to detect the anticipated difference. Participants were randomized by the study statistician at Washington University using a computer generated permuted-block randomization stratified by gender to assign treatment arms. All results are based on intention to treat analysis. Descriptive statistics are reported as frequencies and percentages for categorical variables, means and standard deviations (SDs) for continuous variables, and medians and inter-quartile ranges (IQRs) for skewed continuous variables. Comparisons of TEAEs by treatment arm were performed using chi-square tests or Fisher’s exact tests for categorical variables and Kruskal-Wallis tests for continuous variables. Baseline characteristics considered in our analysis included age (in years), sex, body mass index (BMI, kg/m^2^), any baseline abnormality, and baseline Mf count (dichotomized as greater than or equal to 40 Mf/ml versus less than 40 Mf/ml). Multivariable logistic regression analyses were used to compare odds of any TEAEs by treatment arm, after controlling for baseline covariates. Results were reported as odds ratios (ORs) and 95% confidence intervals (CIs). All analyses were performed in SAS version 9.4 (SAS Institute, Cary, NC) and p-values < 0.05 were considered statistically significant.

### Outcomes

This paper focuses on the safety and tolerability of the drug treatments. Pharmacokinetics and efficacy outcomes will be reported separately.

## Results

**Fig 2:**
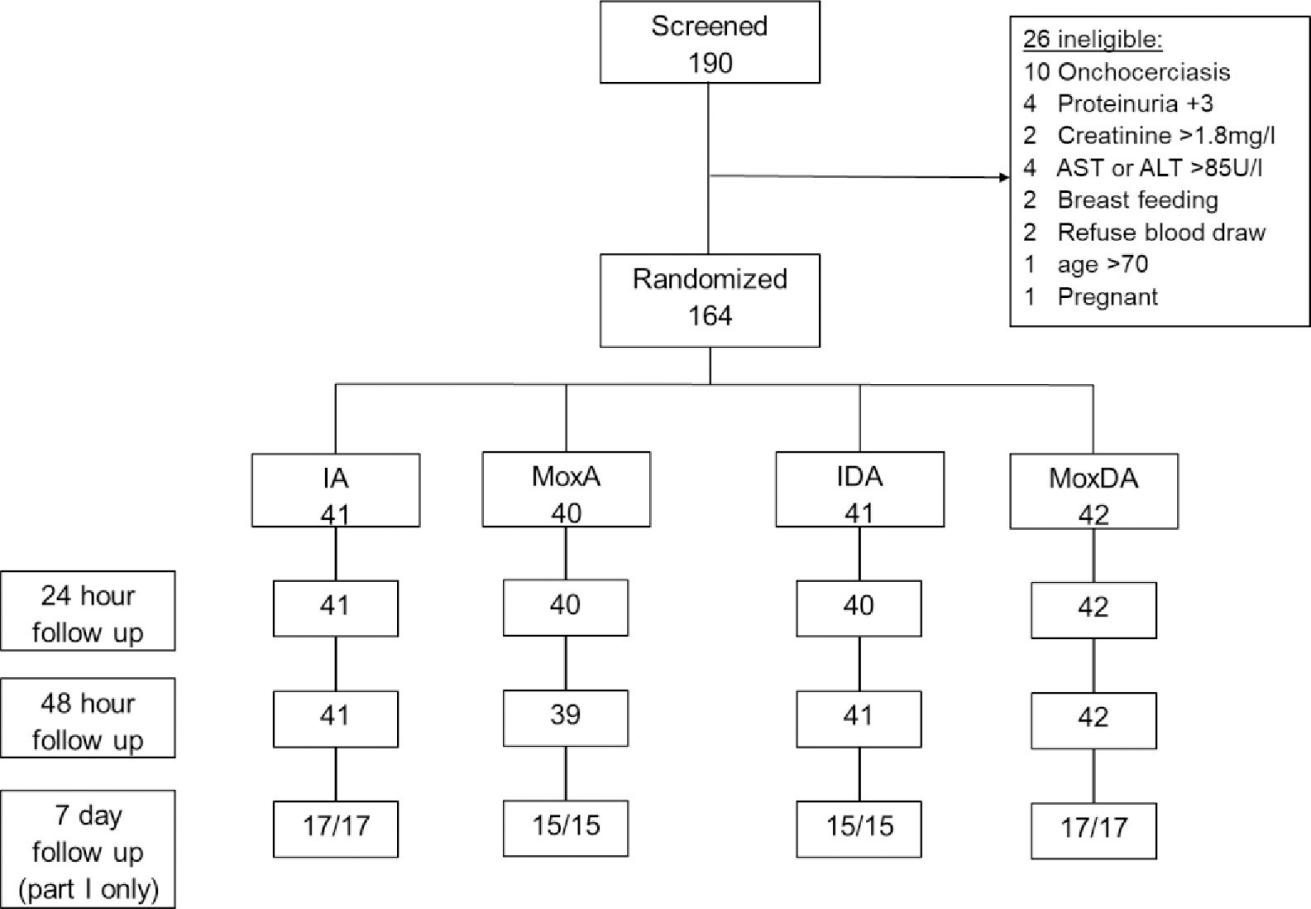
Screening and randomization of study participants (consort diagram).

Community surveys conducted by PNL-SGL of over 9,000 people in the study area between 2018–2020 identified nearly 200 people with at least 3 *W*. *bancrofti* Mf on a 60-μL nocturnal thick blood smear (suggesting they would likely have at least 40 Mf/ml by filtration). One hundred ninety people underwent study screening, of which 164 met all eligibility requirements, were randomized and treated (Fig 2). Seventy-two participants were enrolled in part 1 (intensive inpatient safety monitoring) and the remaining 92 in part 2 (outpatient monitoring). Active AE assessments at 24 and 48 hours were completed for 163 participants (99%) and for all 72 people enrolled in part I on day 7. Baseline demographics of each arm are shown in Table 1. Baseline abnormalities were common with 88 (53%) participants reporting subjective symptoms or having mild vital signs or lab abnormalities prior to treatment.

**Table 1:** Baseline characteristics of enrolled participants.

Variable	All	IA	MoxA	IDA	MoxDA
N	164	41	40	41	42
Age (years), mean ± SD	37.3 ±± 11	35.3 ± 10	35.7 ± 11.1	38.6 ± 11.9	39.3 ± 10.8
Female	15 (9.1%)	4 (9.8%)	3 (7.5%)	4 (9.8%)	4 (9.5%)
BMI (kg/m2), mean ± SD	21.9 ± 2.4	22.3 ± 2.5	21.8 ± 2.7	21.8 ± 2.7	21.6 ± 1.8
Baseline Mf count, median (IQR)	125 (30.5, 327.5)	189 (33, 423)	95.5 (6.5, 210)	133 (61, 391)	107.5 (55, 248)

Eighty-seven of 164 (53%) participants experienced at least 1 TEAE in the seven days post-treatment, including 54% of participants in the IA arm, 60% in MoxA, 44% in IDA and 55% in MoxDA arms (p = 0.530). About one third of participants experienced more than one TEAE; 41%, 32%, 34% and 35% for IA, MoxA, IDA and MoxDA, respectively (p = 0.848). The mean number of TEAEs among those with any TEAE were 3.0, 2.0, 2.9, and 2.6 after IA, MoxA, IDA, or MoxDA, respectively (p = 0.197). There were no serious adverse events in any of the treatment arms. There was no difference in baseline characteristics between those who experienced TEAEs and those who did not (S1 Table). There was no difference in TEAEs for those with ≥ 40 Mf/ml and those with fewer than 40 Mf/ml at baseline (S2 Table).

Among those experiencing subjective AEs, muscle or joint pain, headache, abdominal pain and diarrhea were most common. Scrotal pain or lymph node swelling occurred in six persons treated with IDA or MoxDA, but not after treatment with IA or MoxA (Tables 2 and 3). All subjective TEAEs were mild and resolved within 48 hours. None required intervention. Among those experiencing objective TEAEs, four individuals had fever, 3 grade 1 and one grade 2. Proteinuria was seen in 8.5% and hematuria in 11% of participants with no difference by treatment arm. Mild (grade 1) creatinine increase was seen in 17% of participants. One participant had grade 2 proteinuria, 7 had grade 2 hematuria and 4 had grade 3 hematuria. These TEAEs occurred in all treatment groups.

**Table 2:** Summary of treatment emergent adverse events (TEAE), overall and by study arm.

Value	Total	IA	MoxA	IDA	MoxDA
Any baseline abnormality	88 (53.7%)	20 (48.8%)	23 (57.5%)	25 (61%)	20 (47.6%)
Any TEAE	87 (53%)	22 (53.7%)	24 (60%)	18 (43.9%)	23 (54.8%)
Subjective TEAE	60 (36.6%)	16 (39%)	17 (42.5%)	12 (29.3%)	15 (35.7%)
Objective TEAE	65 (39.6%)	17 (41.5%)	14 (35%)	15 (36.6%)	19 (45.2%)
Multiple TEAEs	59 (36%)	17 (41.5%)	13 (32.5%)	14 (34.1%)	15 (35.7%)
Average # of TEAEs (among those with any TEAE)	2.6	3.0	2.0	2.9	2.6
Any Grade 2 TEAE	14 (8.5%)	3 (7.3%)	1 (2.5%)	6 (14.6%)	4 (9.5%)

Frequencies reflect the number of individuals reporting each AE term, regardless of the number of times that AE term was reported by the individual.

Based on results of multivariable analyses, there were no differences in TEAE after adjusting for age, sex, BMI category, treatment arm, baseline abnormality or baseline Mf count.

**Table 3:** Summary of subjective treatment emergent adverse events (TEAE), overall and by study arm.

AE Term	Total	IA	MoxA	IDA	MoxDA
Abdominal Pain	15 (9.1%)	7 (17.1%)	2 (5%)	4 (9.8%)	2 (4.8%)
Cough	4 (2.4%)	2 (4.9%)	0 (0%)	0 (0%)	2 (4.8%)
Diarrhea	15 (9.1%)	5 (12.2%)	3 (7.5%)	3 (7.3%)	4 (9.5%)
Difficulty Breathing (wheezing/dyspnea)	1 (0.6%)	0 (0%)	0 (0%)	0 (0%)	1 (2.4%)
Dizziness, giddiness, or fainting	11 (6.7%)	5 (12.2%)	1 (2.5%)	1 (2.4%)	4 (9.5%)
Fatigue	4 (2.4%)	1 (2.4%)	2 (5%)	0 (0%)	1 (2.4%)
Headache	21 (12.8%)	9 (22%)	4 (10%)	3 (7.3%)	5 (11.9%)
Itching skin	11 (6.7%)	2 (4.9%)	7 (17.5%)	0 (0%)	2 (4.8%)
Muscle or joint pain	26 (15.9%)	5 (12.2%)	11 (27.5%)	5 (12.2%)	5 (11.9%)
Nausea	9 (5.5%)	3 (7.3%)	1 (2.5%)	3 (7.3%)	2 (4.8%)
Other	4 (2.4%)	0 (0%)	0 (0%)	3 (7.3%)	1 (2.4%)
Swollen or painful nodules	1 (0.6%)	0 (0%)	0 (0%)	1 (2.4%)	0 (0%)
Testicular or scrotal pain	6 (3.7%)	0 (0%)	0 (0%)	3 (7.3%)	3 (7.1%)
Vomiting	1 (0.6%)	0 (0%)	0 (0%)	1 (2.4%)	0 (0%)

Frequencies reflect the number of individuals reporting each AE term, regardless of the number of times that AE term was reported by the individual

Based on results of multivariable analyses, there were no differences in TEAE after adjusting for age, sex, BMI category, treatment arm, baseline abnormality or baseline Mf count.

Overall, 8.5% of participants experienced a grade 2 TEAE, and TEAEs did not differ significantly between groups, occurring in 3/42 (7.3%) after IA, 1/40 (2.5%) after MoxA, 6/41 (14.6%) after IDA, and 4/42 (9.5%) after MoxDA (p = 0.267). Most grade 2 TEAEs had resolved by day 7. One person was lost to follow up and 2 with persistence at 7 days had resolution at the time of a repeat visit later in the study).

## Discussion

This is the first evaluation of the safety and tolerability of moxidectin in combination with albendazole (with or without DEC) for the treatment of LF in people with *W*. *bancrofti* microfilaremia. It showed that these combinations are safe, with similar safety profiles to the current WHO recommended treatments of IA and IDA.

Mild to moderate systemic TEAEs are common immediately following LF treatment, and these are primarily related to host responses to dying Mf. They generally occur in the first 24–48 hours after treatment and resolve in 1–2 days [8,9]. Death of adult worms can lead to delayed inflammatory reactions with new or worsened lymphedema, hydroceles and scrotal pain. These reactions often start several days to 1 week after treatment and can last for several weeks; rates and severity of TEAEs following treatment increase with increasing Mf counts [10]. TEAEs in uninfected persons treated for LF can include nausea, vomiting or diarrhea. These may be directly drug related [11, 12] or due to effects of the drugs on intestinal helminths. For macrocyclic lactones (ivermectin and moxidectin), adverse events in healthy, uninfected volunteers may include mild and transient CNS effects (headache, dizziness), potentially related their ability to bind mammalian GABA receptors, and a safety profile similar to placebo [13–18].

While albendazole affects the viability and reproductive capacity of adult filarial worms, it does not rapidly kill Mf or adult worms, and it does not commonly cause TEAEs [19]. DEC is both a microfilaricidal and macrofilaricidal drug, so TEAEs after DEC treatment can be related to the death of both Mf and adult worms. Swollen lymph nodes and scrotal pain in this study were only seen in those receiving DEC containing regimens, which is consistent with the localization of the adult worms in lymphatic vessels. IVM is a microfilaricidal agent that does not kill adult worms in the dose used in this study. Combinations IA and MoxA have recently been compared for use against soil transmitted helminths, particularly *Trichuris trichiura* [20], headache and abdominal pain were more common in that study than in the present study, but the authors did not report scrotal pain or swollen lymph nodes, and they also did not find any difference between IVM or Mox containing regimens. In our study we did not test for intestinal helminths and so cannot comment on their possible contribution to our findings.

Limitations of our study include the small sample size per arm, limited enrollment of women and the lack of children. Prior studies have observed different frequencies of TEAEs between men and women treated for LF [5]. The paucity of women in this study precludes a valid comparison. Every effort was made to include more women, however, although approximately equal numbers of women and men were screened for inclusion, very few women met FTS and Mf inclusion criteria. This is most likely due to higher participation of women in prior rounds of MDA in this area. Children were not included in this study, because moxidectin is not currently approved for persons <12 years of age. Additional studies will be needed to assess the safety of moxidectin in subjects with LF in that age group. Also, larger studies will be needed to confirm the safety results observed in this study, which was not powered to detect rare TEAEs.

Another limitation of the study was our exclusion of some individuals who would normally be included in MDA. In this study we excluded those with onchocerciasis, since it is a contraindication to receiving DEC. We excluded persons >70 years old to reduce the risk of loss to follow-up due to frailty or other medical complications over the course of three years of follow up. This, and the small size of the study, have some limiting effect on the generalizability of our findings. However, it is unlikely that inclusion of these groups would have significantly altered the results, as moxidectin has previously been shown to be safe in individuals with onchocerciasis [7], and there is no reason to suspect that the safety profile of single-dose moxidectin would be different in those aged >70 years. The small size of the study, which is powered for the primary efficacy endpoint, is a limitation that will need to be addressed in large safety studies, should moxidectin combination therapy prove more effective than ivermectin-based therapy for LF.

In conclusion, moxidectin combination treatments for LF were safe and well tolerated in this study. Moxidectin combination treatments seem to have the same TEAE profile as ivermectin combinations that are widely used in MDA programs to eliminate LF. Many areas in Africa are co-endemic for LF and onchocerciasis, and moxidectin is more effective than ivermectin for clearing *O*. *volvulus* Mf from the skin. Thus, if moxidectin combination treatments are more effective than ivermectin combination treatments for treatment of LF, they may prove useful accelerating LF elimination in sub-Saharan Africa.

## Supporting information

S1 Table Baseline demographic characteristics whether or not an individual experienced any treatment emergent adverse AE. (TIF)

S2 Table Summary of treatment emergent adverse events (TEAE) by baseline Mf level. (TIF)

S1 Protocol Supporting information: Protocol. (DOCX)

S1 Consort check list Consort check list. (PDF)
