# Supplementary figures and images for: Safety and tolerability of moxidectin and ivermectin combination treatments for lymphatic filariasis in Côte d’Ivoire: A randomized controlled superiority study
Source: PLoS Negl Trop Dis. 2023 Sep 18;17(9):e0011633. doi: 10.1371/journal.pntd.0011633 (PMC10538700; doi:10.1371/journal.pntd.0011633)

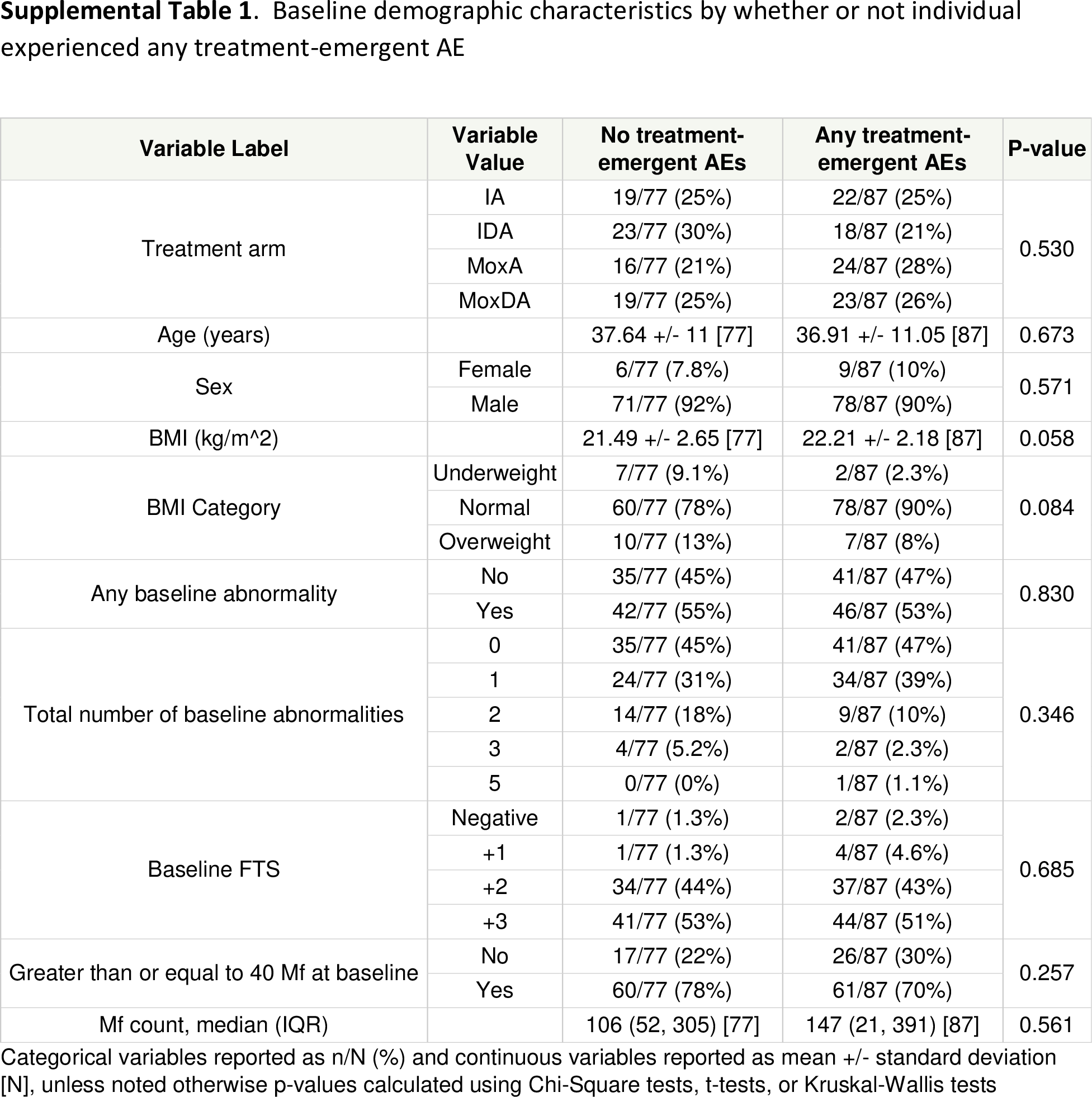

Supplement: S1 Table — (TIF) [file pntd.0011633.s001.tif]

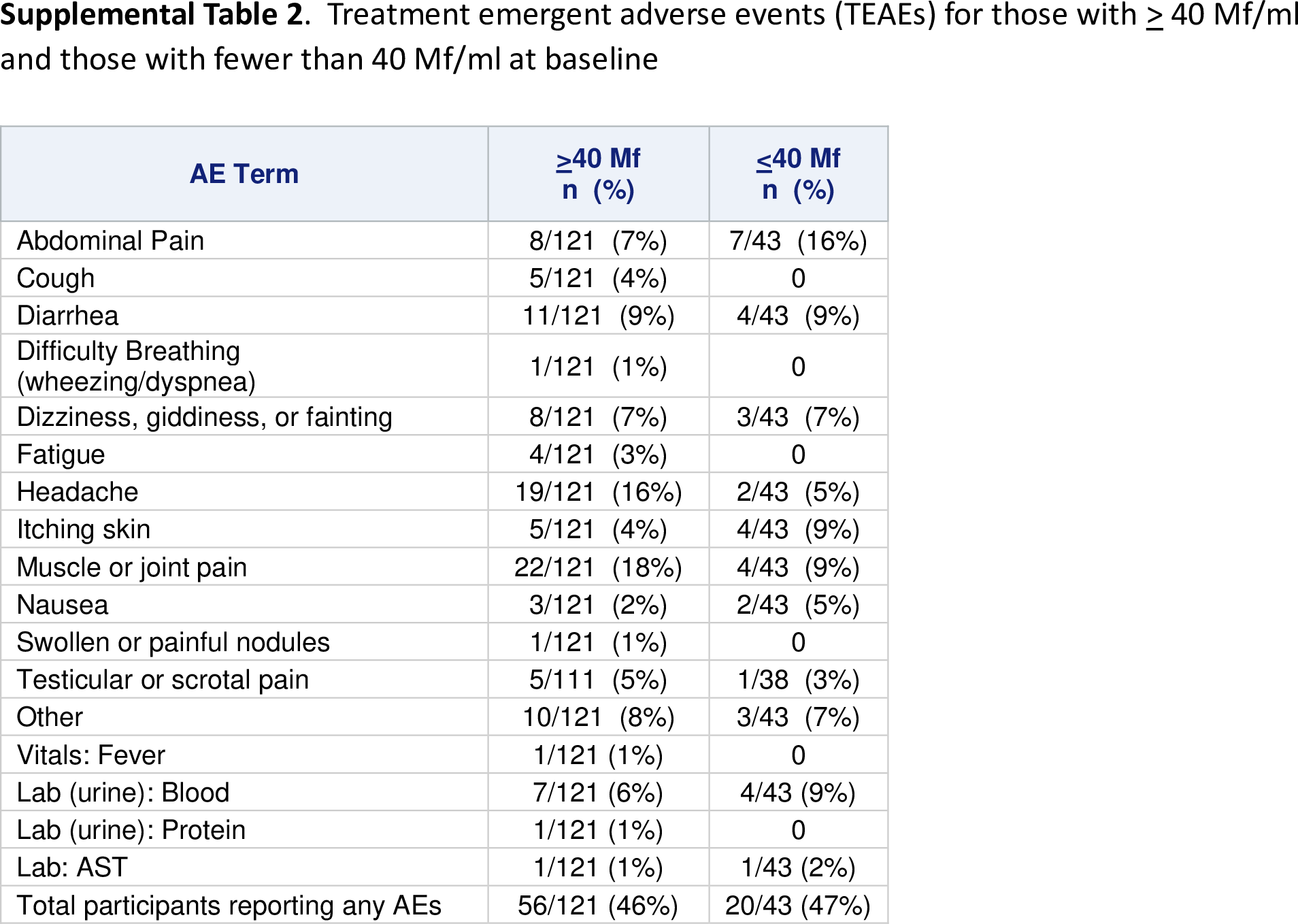

Supplement: S2 Table — (TIF) [file pntd.0011633.s002.tif]
