# Supplementary material for: Safety and tolerability of moxidectin and ivermectin combination treatments for lymphatic filariasis in Côte d’Ivoire: A randomized controlled superiority study
Source: PLoS Negl Trop Dis. 2023 Sep 18;17(9):e0011633. doi: 10.1371/journal.pntd.0011633 (PMC10538700; doi:10.1371/journal.pntd.0011633)
Supplement: S1 Protocol — (DOCX) [file pntd.0011633.s003.docx]

**A Clinical Trial to Assess the Safety and Efficacy of Moxidectin Combination Treatments vs. Ivermectin Combination Treatments for Bancroftian Filariasis**

**Protocol Number:** TBD

**National Clinical Trial (NCT) Identified Number:** TBD

**Principal Investigators**

| Toki Pascal Gabo, MD | Centre Hospitalier Régional d’Agboville, Côte d’Ivoire |
| --- | --- |
| Benjamin Koudou, PhD | Centre Suisse de Recherches Scientifiques, Côte d’Ivoire |
| Catherine M. Bjerum, MD, MPH | Case Western Reserve University, USA |
| Philip J. Budge, MD, PhD | Washington University, USA |

**Co Investigators**

| Allassane Ouattara | Centre Suisse de Recherches Scientifiques, Côte d’Ivoire |
| --- | --- |
| Aboulaye Méité | Programme National de la Lutte Contre la Schistosomiase, les Geohelminthiases et la Filariose Lymphatique, Côte d’Ivoire (PNL-SGF) |
| Gary J. Weil, MD | Washington University, USA |
| Peter Fischer, PhD | Washington University, USA |
| Christopher L. King, MD, PhD | Case Western Reserve University, USA |

**Sponsor:** Washington University in St. Louis

**Funded by:** Bill and Melinda Gates Foundation

**Version Number: v.2.2**

1 June 2021

**Summary of Changes from Previous Version:**

| **Affected Section(s)** | **Summary of Revisions Made** | **Rationale** |  |
| --- | --- | --- | --- |
| 8.1 | Will collect urine and store samples at -20⁰C for further studies to identify Wuchereria biomarkers | Identification of new non-invasive biomarkers for detecting W. bancrofti |  |
|  |  |  |  |
|  |  |  |  |
|  |  |  | |
|  |  |  | |
|  |  |  | |

Table of Contents

[STATEMENT OF COMPLIANCE 2](#_Toc25685335)

[1 PROTOCOL SUMMARY 2](#_Toc25685336)

[1.1 Synopsis 2](#_Toc25685337)

[1.2 Schema 2](#_Toc25685338)

[1.3 Schedule of Activities (SoA) 2](#_Toc25685339)

[2 INTRODUCTION 2](#_Toc25685340)

[2.1 Study Rationale 2](#_Toc25685341)

[2.2 Background 2](#_Toc25685342)

[2.3 Risk/Benefit Assessment 2](#_Toc25685343)

[2.3.1 Known Potential Risks 2](#_Toc25685344)

[2.3.2 Known Potential Benefits 2](#_Toc25685345)

[2.3.3 Assessment of Potential Risks and Benefits 2](#_Toc25685346)

[3 OBJECTIVES AND ENDPOINTS 2](#_Toc25685347)

[4 STUDY DESIGN 2](#_Toc25685348)

[4.1 Overall Design 2](#_Toc25685349)

[4.2 Scientific Rationale for Study Design 2](#_Toc25685350)

[4.3 Justification for Dose 2](#_Toc25685351)

[4.4 End of Study Definition 2](#_Toc25685352)

[5 STUDY POPULATION 2](#_Toc25685353)

[5.1 Inclusion Criteria 2](#_Toc25685354)

[5.2 Exclusion Criteria 2](#_Toc25685355)

[5.3 Screen Failures 2](#_Toc25685356)

[5.4 Strategies for Recruitment and Retention 2](#_Toc25685357)

[6 STUDY INTERVENTION 2](#_Toc25685358)

[6.1 Study Intervention(s) Administration 2](#_Toc25685359)

[6.1.1 Study Intervention Description 2](#_Toc25685360)

[6.1.2 Dosing and Administration 2](#_Toc25685361)

[6.2 Preparation/Handling/Storage/Accountability 2](#_Toc25685362)

[6.2.1 Acquisition and accountability 2](#_Toc25685363)

[6.2.2 Formulation, Appearance, Packaging, and Labeling 2](#_Toc25685364)

[6.2.3 Product Storage and Stability 2](#_Toc25685365)

[6.3 Measures to Minimize Bias: Randomization and Blinding 2](#_Toc25685366)

[6.4 Study Intervention Compliance 2](#_Toc25685367)

[6.5 Concomitant Therapy 2](#_Toc25685368)

[7 PARTICIPANT DISCONTINUATION/WITHDRAWAL 2](#_Toc25685369)

[7.1 Participant Discontinuation/Withdrawal from the Study 2](#_Toc25685370)

[7.2 Lost to Follow-Up 2](#_Toc25685371)

[8 STUDY ASSESSMENTS AND PROCEDURES 2](#_Toc25685372)

[8.1 Efficacy Assessments 2](#_Toc25685373)

[8.2 Safety and Other Assessments 2](#_Toc25685374)

[8.3 Adverse Events and Serious Adverse Events 2](#_Toc25685375)

[8.3.1 Definition of Adverse Events (AE) 2](#_Toc25685376)

[8.3.2 Definition of Serious Adverse Events (SAE) 2](#_Toc25685377)

[8.3.3 Classification of an Adverse Event 2](#_Toc25685378)

[8.3.4 Time Period and Frequency for Event Assessment and Follow-Up 2](#_Toc25685379)

[8.3.5 Adverse Event Reporting 2](#_Toc25685380)

[8.3.6 Serious Adverse Event Reporting 2](#_Toc25685381)

[8.3.7 Reporting of Pregnancy 2](#_Toc25685382)

[8.4 Unanticipated Problems 2](#_Toc25685383)

[8.4.1 Definition of Unanticipated Problems (UP) 2](#_Toc25685384)

[8.4.2 Unanticipated Problem Reporting 2](#_Toc25685385)

[9 STATISTICAL CONSIDERATIONS 2](#_Toc25685386)

[9.1 Statistical Hypotheses 2](#_Toc25685387)

[9.2 Sample Size Determination 2](#_Toc25685388)

[9.3 Populations for Analyses 2](#_Toc25685389)

[9.4 Statistical Analyses 2](#_Toc25685390)

[9.4.1 General Approach 2](#_Toc25685391)

[9.4.2 Analysis of the Primary Efficacy Endpoint(s) 2](#_Toc25685392)

[9.4.3 Analysis of the Secondary Endpoint(s) 2](#_Toc25685393)

[9.4.4 Safety Analyses 2](#_Toc25685394)

[9.4.5 Baseline Descriptive Statistics 2](#_Toc25685395)

[9.4.6 Planned Interim Analyses 2](#_Toc25685396)

[9.4.7 Sub-Group Analyses 2](#_Toc25685397)

[10 SUPPORTING DOCUMENTATION AND OPERATIONAL CONSIDERATIONS 2](#_Toc25685398)

[10.1 Regulatory, Ethical, and Study Oversight Considerations 2](#_Toc25685399)

[10.1.1 Informed Consent Process 2](#_Toc25685400)

[10.1.2 Study Discontinuation and Closure 2](#_Toc25685401)

[10.1.3 Confidentiality and Privacy 2](#_Toc25685402)

[10.1.4 Future Use of Stored Specimens and Data 2](#_Toc25685403)

[10.1.5 Key Roles and Study Governance 2](#_Toc25685404)

[10.1.6 Safety Oversight 2](#_Toc25685405)

[10.1.7 Clinical Monitoring 2](#_Toc25685406)

[10.1.8 Quality Assurance and Quality Control 2](#_Toc25685407)

[10.1.9 Data Handling and Record Keeping 2](#_Toc25685408)

[10.1.10 Protocol Deviations 2](#_Toc25685409)

[10.1.11 Publication and Data Sharing Policy 2](#_Toc25685410)

[10.2 Abbreviations 2](#_Toc25685411)

[11 REFERENCES 2](#_Toc25685412)

# STATEMENT OF COMPLIANCE

The trial will be conducted in accordance with International Conference on Harmonisation Good Clinical Practice (ICH GCP). All personnel involved in the conduct of this study have completed Human Subjects Protection and ICH GCP Training.

The protocol, informed consent forms, recruitment materials, and all participant materials will be submitted to the Comité National d’Ethique et de la Recherche (CNER) in Côte d’Ivoire and to the Washington University institutional review board (IRB), for review and approval. Approval of both the protocol and the consent form must be obtained before any participant is enrolled. Any amendment to the protocol will require review and approval by the IRB and CNER before the changes are implemented to the study.

# PROTOCOL SUMMARY

## Synopsis

| **Title:** | A Clinical Trial to Assess the Safety and Efficacy of Moxidectin Combination Treatments vs. Ivermectin Combination Treatments for Bancroftian Filariasis |
| --- | --- |
| **Study Description:** | This is an interventional, randomized, parallel-assignment, open-label, clinical trial with four treatment arms:   1. **IA**: ivermectin (IVM) plus albendazole (ABZ) 2. **MoxA**: moxidectin (Mox) plus ABZ 3. **IDA**: IVM plus diethylcarbamazine (DEC) plus ABZ 4. **MoxDA**: Mox plus DEC plus ABZ   The IA group will receive IA annually. Participants in the other groups will receive the assigned treatment at baseline and again at 24 months, if microfilaremic. If IA is superior to MoxA at 12 months, the MoxA group will switch to annual IA treatment for the duration of the study. |
| **Objectives:** | **Primary Objective:** To determine whether Mox combination therapies are superior to IVM combination therapies for achieving sustained clearance of *Wuchereria bancrofti* microfilaremia |
|  | **Secondary Objectives:**   1. To compare the efficacy of Mox combination therapies to IVM combination therapies for reducing *W. bancrofti* microfilariae (Mf) counts (change relative to baseline). 2. To compare the efficacy of Mox combination therapies to IVM combination therapies for reducing the viability of *W. bancrofti* adult worms, as assessed by change in circulating filarial antigen (CFA) levels and % inactivation of adult worm nests visible by ultrasound. 3. To compare the safety and tolerability of Mox combination therapies to IVM combination therapies in men and women with *W. bancrofti* infection. 4. To document the pharmacokinetic parameters of Mox in *W. bancrofti*-infected adults and to determine whether Mox affects the peak levels and clearance of DEC and ABZ in individuals with *W. bancrofti* infection, compared to IVM. |
| **Endpoints:** | **Primary Outcomes:**  The proportion of participants in each study arm with complete clearance of *W. bancrofti* microfilaremia at 12 months after treatment (IA vs. MoxA comparison) or 24 months after treatment (IDA vs. MoxDA comparison).  **Key Secondary Outcomes:**  **Efficacy Outcomes**   1. The proportion of participants in each study arm with complete clearance of *W. bancrofti* microfilaremia at 6, 12, 24, and 36 months after treatment 2. Reduction in Mf counts (relative to baseline) at 6, 12, and 24 months 3. Reduction in CFA levels (relative to baseline) at 6, 12, and 24 months 4. Inactivation of adult worm nests as assessed by ultrasound at 6, 12, and 24 months after treatment   **Safety Outcome**   1. Frequency and severity of adverse events (AEs) during the first 7 days after treatment   **Pharmacokinetics Outcome (PK)**   1. Plasma levels of treatment drugs/metabolites post-treatment |
| **Study Population:** | Adults (men and women age 18-70 years) with *W. bancrofti* infection and > 40 Mf/mL venous blood, residing in or near Agboville district, Côte d’Ivoire |
| **Phase:** | Phase 3 |
| **Description of Sites/Facilities Enrolling Participants:** | This trial will be conducted at the Centre de Recherche de Filariose Lymphatique d’Agboville (CRFLA), located at the Centre Hospitalier Regional (CHR) d’Agboville, Cote d’Ivoire and in the surrounding communities. It will be done in coordination with the national LF elimination program’s regional assessment of LF transmission. This trial will be conducted in **two parts*.*** All participants in both parts will be treated with combination therapy for LF and will be followed for 36 months. Part 1 and Part 2 differ in the intensity of AE monitoring and collection of additional blood samples in the first week post-treatment; subsequent follow-up visits (at 6, 12, 24, and 36 months) are the same for both parts.  **Part 1** is a preliminary safety study comprising at least 50 participants (~12 from each arm), who will be treated as inpatients at CRFLA with enhanced AE monitoring and pharmacokinetics (PK) measurements.  **Part 2**, comprising the rest of the study participants, will proceed if no serious safety signals are observed in Part 1 (i.e. significantly more treatment-related severe or serious AEs in participants who have been treated with Mox drug combinations). Part 2 participants will be treated and followed for safety and efficacy as outpatients in their home villages, in or near Agboville. |
| **Description of Study Intervention:** | All participants will receive an oral dose of one of the following drug combinations:   1. IVM 200 µg/kg + ABZ 400 mg (**IA**) 2. Mox 8 mg + ABZ 400 mg (**MoxA**) 3. IVM 200 µg/kg + DEC 6mg/kg + ABZ 400 mg (**IDA**) 4. Mox 8 mg + DEC 6mg/kg + ABZ 400 mg (**MoxDA**)   Arm 1 will receive IA annually. The other arms will receive treatment at enrollment and following the 24-month assessment, if Mf positive at 24 months. |
| **Study Duration:** | 42 Months |
| **Participant Duration:** | 36 Months |

## Schema


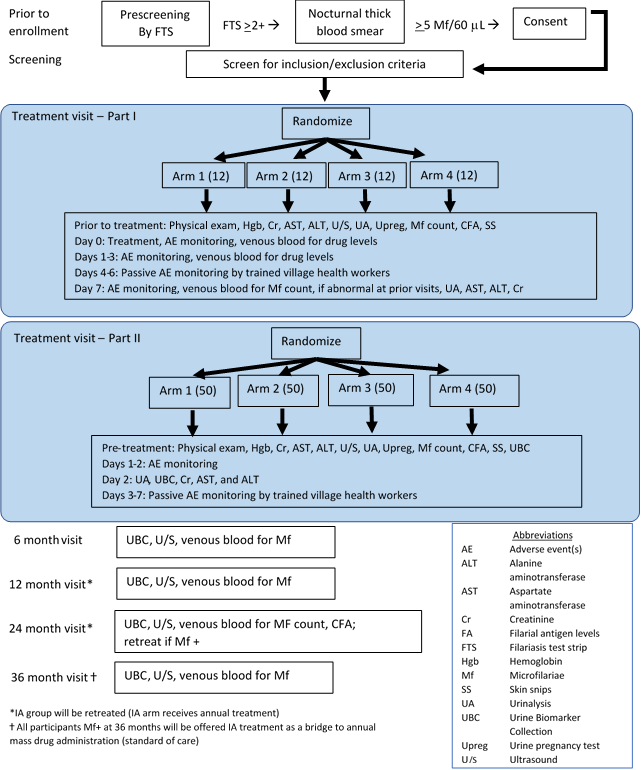


Notes

1. Prescreening by FTS and nocturnal Mf counts will be done by PNL-CSGF as part of their usual public health activities.
2. Drug levels will be assessed only in Part 1
3. Part 1 and Part 2 differ in the number and nature of assessments during the first 7 days only. All follow-up assessments are the same for both parts. See the next section (Schedule of Activities) for details.

## Schedule of Activities (SoA)

|  | Screening / Randomization | Day 0^1^ | | | | | | | Day 1^1^ | | | | Day 2^1^ | | Day 3^1,2^ | Day 7 | 6 months | 12 months | 18 months | 24 months | 36 months |
| --- | --- | --- | --- | --- | --- | --- | --- | --- | --- | --- | --- | --- | --- | --- | --- | --- | --- | --- | --- | --- | --- |
| **Procedures** | Baseline | 0700 (0 hr) | 0900 (2 hr) | 1000 (3 hr) | 1100 (4 hr) | 1300 (6 hr) | 1900 (12 hr) | 0100 (18 hr)^3^ | 0700 (24 hr) | 1300 (30 hr) | 1900 (36 hr) | 0100 (42 hr)^3^ | 0700 (48 hr) | 1900 (60 hr) | 0700 (72 hr) |  |  |  |  |  |  |
| Informed consent | X |  |  |  |  |  |  |  |  |  |  |  |  |  |  |  |  |  |  |  |  |
| Physical exam^4^ | X |  |  |  |  | x^4^ | x^4^ | x^4^ | x^4^ | x^4^ | x^4^ | x^4^ | x^4^ | x^4^ | x^4^ |  |  |  |  |  |  |
| Vital signs^5^ | X | X |  |  |  | X | X | X | X | X | x | x | X | x | X | x^6^ |  |  |  |  |  |
| AE assessment^5^ | X | X |  |  |  | x | X | x | X | x | x | x | x | x | x | x^6^ |  |  |  |  |  |
| Mf count (filtration) | x |  |  |  |  |  |  |  |  |  |  |  |  |  |  | x | x | x |  | x | x |
| Filariasis test strip (FTS) | X |  |  |  |  |  |  |  |  |  |  |  |  |  |  |  | X | X |  | X | X |
| Hgb | x |  |  |  |  |  |  |  |  |  |  |  |  |  |  |  |  |  |  |  |  |
| Cr, AST, ALT | x |  |  |  |  |  |  |  | x |  |  |  | x |  | x | x^7^ |  |  |  |  |  |
| Urinalysis | x |  |  |  |  |  |  |  | x |  |  |  | x |  | x | x^7^ |  |  |  |  |  |
| Urine biomarker collection | x |  |  |  |  |  |  |  |  |  |  |  | x |  |  |  | x | x |  | x | x |
| Urine pregnancy test | x |  |  |  |  |  |  |  |  |  |  |  |  |  |  |  |  | X^8^ |  | X^8^ |  |
| Skin snip | x |  |  |  |  |  |  |  |  |  |  |  |  |  |  |  |  |  |  |  |  |
| Plasma drug levels | x |  | x | x | x | x | x |  | x |  |  |  | x |  |  |  |  |  |  |  |  |
| Circulating filarial antigen | x |  |  |  |  |  |  |  |  |  |  |  |  |  |  | x | x | x |  | x | x |
| Ultrasound (males only) | x |  |  |  |  |  |  |  |  |  |  |  |  |  |  |  | x | x |  | x | x |
| Study drug administration |  | x |  |  |  |  |  |  |  |  |  |  |  |  |  |  |  | x^9^ |  | x^9^ | x^9^ |
| Community visit to share results |  |  |  |  |  |  |  |  |  |  |  |  |  |  |  |  |  | X | x | X | X |
| Shaded fields indicate assessments that are unique to Part 1 (inpatient monitoring / pharmacokinetics). All other assessments apply to both Part 1 and Part 2.  Notes   1. Times of day listed for days 0, 1, 2, and 3 apply only to Part 1. 2. For Part 1, all patients will have a 72 hour assessment. For Part 2, only patients with grade 2 or higher AEs not resolved by day 2 will be visited on day 3. 3. Assessments at 18 and 42 hours (Part 1) may be skipped if patient is sleeping. 4. Targeted physical exam will be performed if any grade 2 or higher AE is observed or if abnormal vital signs are noted. 5. Vital signs will include temperature, pulse rate, respiration rate, and blood pressure. 6. Any participant in Part 1 or Part 2 experiencing grade 2 or higher AE(s) will be followed by the study team until AE severity is <grade 2 (including beyond 7 days if necessary). 7. If post-treatment biochemistries and urinalysis done on day 7 post-treatment (Part 1 only) represent grade 2 or higher AEs, participants will be followed with repeat lab monitoring until AE severity is <grade 2 (including beyond 7 days if necessary) 8. Only participants found to be microfilaremic at 24 months will be re-treated, and only women requiring re-treatment will undergo pregnancy test at 24 months. 9. IA group will receive treatment annually. Individual MoxA, IDA, and MoxDA participants will be retreated at 24 months only if Mf positive. Participants in any group who are Mf positive at 36 months will be offered IA treatment as a bridge back to annual mass drug administration (standard of care).   Abbreviations: Mf = microfilariae, Cr = creatinine, AST = aspartate aminotransferase, ALT = alanine aminotransferase | | | | | | | | | | | | | | | | | | | | | |

# INTRODUCTION

## Study Rationale

The purpose of the current study is to determine whether moxidectin (Mox), will be more effective than ivermectin (IVM), when used in single-dose combination therapies for lymphatic filariasis (LF). Mox was approved in June 2018 by the U.S. FDA for treatment of the related filarial infection, onchocerciasis (1). Its mode of action is similar to that of IVM, and a single treatment dose does not kill adult *Onchocerca volvulus* worms, but is superior to IVM for achieving prolonged clearance of microfilariae (Mf) from the skin (2). No clinical trials have been performed to date to assess the efficacy of Mox (alone or in combination with DEC or ABZ) for treatment of LF. This study will provide the first data on the relative safety of MoxDA and MoxA vs. IDA or IA for treating bancroftian filariasis. This study will also examine whether Mox affects plasma levels of ABZ and/or DEC when given in combination.

## Background

Lymphatic Filariasis (LF) is a deforming and disabling disease caused by the mosquito-borne parasitic nematodes *W. bancrofti*, *Brugia. malayi and B. timori*. Infection causes lymphatic dysfunction leading to hydroceles (in men) and lymphedema that can progress to elephantiasis. Global efforts to eliminate LF transmission have reduced the population at risk for the disease, but LF transmission continues in at least 53 countries with 886 million still at risk (3-5).

The World Health Organization’s (WHO) Global Programme to Eliminate LF (GPELF) includes population-based surveys to determine where LF is endemic (mapping), followed by at least five rounds of annual mass drug administration (MDA) to treat the entire eligible population with antifilarial medications (4, 6). The most recent summary from WHO reported that more than 7 billion doses of MDA medications were distributed between 2000 and 2017 (5), making GPELF the largest MDA-based infectious disease intervention program attempted to date. The medications given during MDA include IVM, DEC, and ABZ.

GPELF recommends the IA combination for LF MDA in areas of Africa where LF is co-endemic with onchocerciasis, twice yearly ABZ alone in areas with co-endemic loiasis, and DEC plus ABZ in all other LF-endemic areas (7). However, triple drug combination therapy with all three medications (IDA) was recently found to be superior to the 2-drug combinations in both Papua New Guinea and Cote d’Ivoire (8-10). IDA triple therapy produces prolonged Mf clearance after only one dose compared to results obtained after either DA or IA. This is a potential "game changer" for GPELF because it suggests that MDA with IDA might achieve interruption of transmission with 3 or fewer rounds of MDA, rather than the 5 or more rounds required using dual drug combinations (11). Large-scale international community safety trials in over 20,000 participants have shown no increase in adverse events (AEs) with IDA compared to DA, and IDA is now recommended by WHO for MDA in areas of the world without onchocerciasis or loiasis who are unlikely to meet the 2020 goals (7).

Onchocerciasis, or "river blindness", caused by *O. volvulus*, is another filarial infection targeted for elimination through MDA. WHO calls for MDA with IVM plus ABZ where LF and onchocerciasis are co-endemic, and IVM alone in areas with only onchocerciasis. DEC is not used for MDA in LF/onchocerciasis co-endemic areas, because it can cause serious ocular AEs in persons with intraocular *O. volvulus* Mf. Neither DEC nor IVM are safe for routine MDA in areas with loiasis (“African eye worm”) caused by *Loa loa*, since severe AEs (SAEs) including encephalopathy and death have occurred in persons with very heavy loiasis (>20,000 *L. loa* Mf/mL in peripheral blood) infections after treatment with either of these medications. This risk of SAEs in loiasis-endemic African countries has delayed effective MDA in these areas and remains a major challenge to LF and onchocerciasis elimination in Africa. Other challenges facing the GPELF include the limited macrofilaricidal activity of current MDA regimens, which necessitates repeated annual rounds of MDA, and the difficulty of achieving high compliance rates for MDA over a period of years (12, 13).

Although Mox is not a macrofilaricidal drug, it likely will work together with DEC and ABZ in a way that is similar to IDA. It is also possible that MoxA will be superior to IA for achieving prolonged Mf clearance with similar efficacy to IDA without requiring DEC. If this is the case, it would be a very significant advance, because then MoxA could be used to eliminate LF in onchocerciasis coendemic areas without having to use DEC. A second reason to test MoxA and MoxDA as treatments for LF is that many participants with LF treated with IDA in the clinical trial conducted in Cote d’Ivoire had only transient clearance of Mf; many participants were microfilaremic 12 and 24 months after treatment (see Rationale for study 1.4, above). This finding makes it important to test whether MoxA or MoxDA are superior to IA or IDA, respectively, for achieving prolonged clearance of *W. bancrofti* microfilaremia in Africa.

**Drug Mechanisms**

**Albendazole (ABZ)**

ALB, a benzimidazole, causes degenerative alterations in the tegument and intestinal cells of the worm by binding to the colchicine-sensitive site of tubulin, thus inhibiting its polymerization or assembly into microtubules. The loss of cytoplasmic microtubules leads to impaired uptake of glucose by larval and adult stages of the parasite, and depletes glycogen stores (14). Degenerative changes in endoplasmic reticulum and mitochondria of the germinal layer, and the subsequent release of lysosomal enzymes result in decreased production of adenosine triphosphate, which is the source of energy required for survival of the helminth. Due to diminished energy production, the parasite is immobilized and eventually dies.

ABZ is poorly absorbed; its absorption is enhanced 4–5-fold when it is ingested with a fatty meal (15). The drug undergoes first-pass metabolism within intestinal mucosal cells; the liver converts most of the drug into the active metabolite, albendazole sulfoxide (ABZSO) (16) . This conversion is catalyzed by microsomal flavin monooxygenase and isoforms of cytochrome P450 (CYP). Large inter-individual variations in serum concentrations of ABZSO are due either to variation in first-pass metabolism or in the rate of absorption of ABZ. ABZSO is approximately 70% bound to serum proteins and has a highly variable half-life.

**Ivermectin (IVM)**

IVM is an avermectin compound of macrocyclic lactones derived from the bacterium *Streptomyces avermitilis* (17). The mechanism by which IVM kills LF microfilariae is not known with certainty, but the drug interferes with glutamate gated ion channels that can affect parasite contractility and release of immunomodulatory molecules by the parasite (18). IVM also has direct effects on the parasite nervous system and muscle function as it enhances strength of inhibitory neurotransmission pathways. Neurotoxicity has not been observed in humans given single dose IVM for LF or other parasitic infections, and the drug has been used to treat many millions of people with LF and onchocerciasis (19). Peak IVM serum concentrations are reached approximately 4-5 hours after administration. The half-life of IVM in various populations ranges from 12 to 56 hours (20). The drug is highly bound to serum proteins (93%), and is converted to 10 metabolites by CYP3A4, with nearly no IVM detectable in the urine. Animal studies demonstrate that most of the cleared drug is found unchanged in the feces; this is likely to be true in humans. There is no evidence of drug:drug interaction between ABZ and IVM (21).

**Diethylcarbamazine (DEC)**

DEC is an anthelminthic drug that is structurally distinct from ABZ and IVM (22). The drug has potent microfilaricidal activity against LF (with differing kinetics relative to IVM, which has greater activity than DEC during the first few weeks following ingestion), and modest to high efficacy in killing of adult worms (ABZ has weaker activity against adult worms, whereas IVM is believed not to affect adult worm viability) (23). DEC inhibits arachidonic acid metabolism by LF, and inducible nitric oxide synthase and the cyclooxygenase pathway may be essential for activity *in vivo* (24). DEC also has anti-inflammatory properties. The drug is rapidly absorbed from the gastrointestinal tract, has a serum half-life of 12 to 14 hours, and is excreted in the urine with little modification by the liver.

**Moxidectin (Mox)**

Mox is a macrocyclic lactone derived from the actinomycete *Streptomyces cyanogriseus*. It is in the same class as ivermectin and has a similar mechanism of action, binding to glutamate-gated chloride channels, GABA receptors, and/or ATP-binding cassette transporters. This leads to increased permeability, influx of chloride ions, hyperpolarization and muscle paralysis. Mox is more lipophilic than ivermectin and distributes widely in tissues. The mean half-life of moxidectin in patients with onchocerciasis is 23.3 days. This prolonged half-life is presumed to be one reason for its increased efficacy, compared to IVM, in achieving sustained clearance of *O. volvulus* Mf (25).

## Risk/Benefit Assessment

### Known Potential Risks

The combination of IVM plus ABZ as used in this trial has a strong safety record; it has been provided as MDA to millions of recipients of MDA for LF (3). As outlined above, the IDA combination is safe when used in areas where loiasis and onchocerciasis are non-endemic (26). AEs are common following treatment of filarial infection and are related to drug efficacy in killing Mf. AEs following MoxA or MoxDA treatment are likely to be similar to those following treatment with IA or IDA. AEs correlate with microfilarial loads in infected individuals and are usually mild and transient. However, SAEs can occur when DEC is given to individuals with onchocerciasis or loiasis, or when ivermectin is given to individuals with loiasis (27). Côte d’Ivoire is non-endemic for loiasis. Onchocerciasis is uncommon in the study area. We will screen all potential participants found to have LF for coexisting onchocerciasis by skin snip (2 biopsy sites—1 from each iliac crest). Individuals with microfiladermia will be excluded from the study.

**Immediate risks**

Self-limited mild-to-moderate systemic AEs are common following treatment of LF with microfilaricidal medications. These include fever, headache, myalgia, fatigue, arthralgia, pruritus, malaise, and gastrointestinal symptoms. Systemic AEs usually resolve within 72 hours of treatment. In a recent clinical trial/pharmacokinetic study of IDA for LF treatment conducted in our target study population found that 100% of 66 participants receiving IDA experienced at least one systemic AE; nine (14%) experienced a grade 2 AE. There were no SAEs and all grade 1 and 2 AEs were self-limited (10).

Some individuals experience localized AEs following treatment that are related to dying adult filarial worms. These AEs can include tender subcutaneous nodules, swollen and tender lymph nodes, testicular swelling and/or pain, epididymitis, hydrocele, and lymphedema. Testicular pain is the most common localized AE, and occurred in less than 0.1% of persons treated in the large-scale community safety studies of DA and IDA (26). We expect similar rates and severity of all AEs following treatment with MoxA or MoxDA.

AE profiles for each of the study medications include:

- **DEC**. The most common side effects are itching and swelling of face, headache, joint pain, unusual tiredness or weakness. These are transient. Less common are dizziness, nausea or vomiting. Fever, painful and tender glands in groin, neck armpits or skin rash can occur, and are usually associated with high burdens of infection as judged by the level of blood Mf.
- **ABZ**. Headache, nausea, stomach pain and vomiting are most common, and usually associated with heavy geohelminth infections. Severe allergic reactions occur very rarely, and include rash, hives, itching, difficulty breathing, tightness in the chest, swelling of the mouth, face, lips, or tongue, dark urine. High dose ABZ occasionally causes reversible reductions in total white blood cell count and sometimes results in elevated liver enzymes, which return to normal with cessation of treatment. These abnormalities are associated with prolonged ABZ treatment courses given for such diseases as neurocysticercosis and hydatid disease, and are highly unlikely following single dose treatment as proposed here (15).
- **IVM**. The most common side effects are diarrhea, dizziness and nausea. Rare side effects include rash, hives, itching, difficulty breathing, tightness in the chest, swelling of the mouth, face, lips, or tongue, eye pain, swelling, or redness, fainting, and tachycardia. Mild decrease in leukocyte counts, elevated liver function tests, and orthostatic hypotension rarely occur. Infrequently, treatment can exacerbate bronchial asthma.
- **Mox.** AEs following Mox treatment of onchocerciasis in 2 clinical trials were similar to those seen with IVM, including eosinophilia, pruritus, musculoskeletal pain, headache, lymphocytopenia, tachycardia, rash, abdominal pain, hypotension, fever, and leukocytosis. Hyperbilirubinemia and transaminase elevations above 5x the normal limit were rare, but more common with Mox than IVM (28).

**Long-range risks**

As described above, mild to moderate, self-limited AEs following LF treatment are common. There are no known late-onset AEs following single-dose therapy for LF, but there is a theoretical risk of fetal toxicity / teratogenicity in pregnant women, which is why they will be excluded from the study.

### Known Potential Benefits

Individual subjects will benefit from treatment of their W. bancrofti infection, which may decrease their risk of developing lymphedema and eventually elephantiasis. Treatment will decrease microfilaremia and thereby decrease the risk that participants serve as a reservoir for infection that can be transmitted to others in the community via mosquitoes. Participants with intestinal helminths may also benefit from the deworming effects of the study medications; those receiving IVM or Mox may also benefit from the effects of those medications on scabies or lice infestations.

This study has potential public health benefits, since millions living in LF-endemic areas worldwide stand to benefit if new MDA combinations accelerate LF elimination.

### Assessment of Potential Risks and Benefits

All treatments that effectively clear Mf induce transient AEs in infected individuals. These AEs are self-limited and of short duration. Benefits of treatment include reducing the risk of long-term disability and of perpetuating disease transmission.

As outlined above, to minimize the risk of severe or serious AEs following treatment (particularly if treated with either combination including DEC), we will exclude persons with onchocerciasis.

# OBJECTIVES AND ENDPOINTS

| OBJECTIVES | ENDPOINTS | JUSTIFICATION FOR ENDPOINTS |
| --- | --- | --- |
| Primary |  |  |
| To determine whether Mox combination therapies are superior to IVM combination therapies for achieving sustained clearance of W. bancrofti microfilaremia. | The proportion of participants in each study arm with complete clearance of W. bancrofti microfilaremia at 12 months (IA vs. MoxA comparison) or 24 months after treatment (IDA vs. MoxDA comparison). | The primary anticipated benefit of MoxA/MoxDA is prolonged Mf clearance, compared to IA and IDA, respectively. |
| Secondary |  |  |
| **Efficacy Outcomes**   1. To compare the efficacy of Mox combination therapies to IVM combination therapies in reducing W. bancrofti Mf levels 2. To compare the efficacy of Mox combination therapies to IVM combination therapies in reducing the viability of W. bancrofti adult worms   **Safety Outcome**   1. To compare the safety and tolerability of Mox combination therapies to IVM combination therapies in men and women with W. bancrofti infection   **Pharmacokinetic Outcome**   1. To determine the pharmacokinetic parameters of Mox in W. bancrofti infected adults and to determine whether Mox affects peak levels and clearance of DEC and ABZ, compared to IVM | 1a. The proportion of participants in each study arm with complete clearance of W. bancrofti Mf at 6, 12, 24, and 36 months  1b. Percent reduction in Mf counts (relative to baseline) at 6, 12, 24, and 36 months after treatment  2a. Percent reduction in circulating filarial antigen (CFA) levels (relative to baseline), at 6, 12, 24, and 36 months after treatment  2b. Percent inactivation of adult worm nests, as assessed by ultrasound at 6, 12, 24, and 36 months after treatment. Prevalence of worm nests at the same time points will also be assessed.  3. Frequency and severity of AEs during the first seven days after treatment  4a. Plasma DEC, ABZ, IVM, and Mox levels at baseline, 2, 3, 4, 6, 12, 24, and 48 hours post-treatment | 1. Some study arms may not achieve complete Mf clearance; examining percent reduction will provide important data about reductions in Mf levels, which correlates with transmission risk.  2. This will show whether MoxA or MoxDA have any macrofilaricidal activity. CFA is a marker of adult worm viability. U/S at 12 months will show whether MoxA/MoxDA can clear worm nests. Subsequent U/S will assess for the possibility of re-infection  3. AEs following microfilaricidal treatment occur in the first 72 hours, and ~5 days following macrofilaricidal treatment.  4. Effect of co-administration of Mox may affect DEC and/or ABZ levels |
| Tertiary/Exploratory |  |  |
| To determine the identity of the *W. bancrofti* antigens released by treatment | Liquid chromatography/mass spectrometry analysis of W. bancrofti antigens released in response to treatment. | May lead to identification of improved biomarkers for filarial infection |

# STUDY DESIGN

## Overall Design

This study will test the hypothesis that Moxidectin combination therapies are superior to ivermectin combination therapies for achieving sustained clearance of *W. bancrofti* microfilaremia.

This trial is designed as single-site, Phase III, randomized, open-label, masked-observer superiority trial with four treatment arms (IA, MoxA, IDA, and MoxDA) with a primary endpoint of proportion of participants achieving complete clearance of microfilaremia at 12 months (IA vs. MoxA comparison) or 24 months (IDA vs. MoxDA comparison). Block randomization by gender will be used to assign treatment arms. The first group of participants will be treated at Agboville Hospital with inpatient AE monitoring (Part 1). The first 48 participants in Part 1 (12 per arm) will also have collection of post-treatment plasma drug levels for pharmacokinetic analysis (PK study). For Part 1, active AE surveillance will be conducted in the hospital on days 1, 2, and 3, post-treatment, and in the participant’s village of residence on day 7 post-treatment and passive surveillance will be conducted by trained village health workers on days 4-6. An interim safety analysis will take place after Part 1. If no safety concerns are identified, the remainder of the participants will be treated in their home villages, with active AE monitoring on days 1 and 2 post-treatment (Part 2) with passive surveillance by trained village health workers on days 3-7. Any participant in either Part 1 or Part 2 experiencing AEs of grade 2 or higher will be followed until AE severity falls below grade 2. Follow-up assessments for efficacy of treatments for all participants (Parts 1 and 2) will be conducted at 6, 12, 24, and 36 months.

Due to a prolonged delay between pre-screening and the onset of study enrollment (caused in part by the COVID-19 pandemic), participants who had >40 Mf/mL at the time of prescreening may be found to have less than 40 Mf/mL at the time of enrollment. The logistics of transporting participants from the village and the requirement for nocturnal blood collection for Mf counts make it impractical to await results of the baseline Mf count (done the night prior to treatment) before treatment the following morning. The first 48 enrolled participants, including those found to have Mf counts <40/mL at the time of enrollment, will be included in the PK analysis. Because AEs are more likely in participants with high Mf counts, total enrollment in Part 1 will continue until at least 12 participants in each study arm with >40 Mf/mL have undergone inpatient monitoring.

The study includes both safety and efficacy analyses. The safety assessment (Part 1 only) ends 7 days after treatment (unless AEs remain grade 2 or higher). The efficacy assessment (Parts 1 and 2 combined) ends when participants are retested for filarial infection 36 months post-treatment. Participants in the IA arm will receive IA annually (standard of care). Participants in the other arms will receive the assigned treatment at baseline; those found to be microfilaremic at 24 months post-treatment will be retreated with the same treatment received at baseline. If clearance of Mf at 12 months in the IA arm is superior to Mf clearance in the MoxA arm, the MoxA group will be switched to annual IA treatment. Participants with <40 Mf/mL enrolled in part 1 will be included in the PK and safety analyses, and potentially secondary analyses of drug efficacy on adult worms, *but only those with Mf counts of at least 40 Mf/mL will be included in the primary analysis of efficacy outcomes*.

The study design does not currently include stratification, nor do any sub-studies. However, the study may stratify based on pre-treatment Mf levels if high variability among pre-screening Mf counts is observed.

## Scientific Rationale for Study Design

Each of the individual study medications are FDA approved with established dosing recommendations and extensive safety data. This will be a superiority study (MoxA vs. IA and MoxDA vs. IDA) because significant benefit to the GPELF would only be achieved if MoxA is superior to IA, which is currently the standard for LF targeted MDA programs where LF is co-endemic with onchocerciasis. A placebo control would be unethical for treatment of persons with microfilaremia.

## Justification for Dose

Doses for each of the trial medications are established and FDA-approved. DEC, IVM, and ABZ doses given in this study will match GPELF recommendations for LF MDA; Mox doses will match those recommended for onchocerciasis MDA.

## End of Study Definition

The end of the study is defined as completion of the last visit or procedure shown in the SOA in the trial globally. A participant is considered to have completed the study once he or she has completed the last visit (36 month) as shown in the Schedule of Activities (SOA), Section 1.3.

# STUDY POPULATION

## Inclusion Criteria

In order to be eligible to participate in this study, an individual must meet all of the following criteria:

1. Provision of signed and dated informed consent form
2. Male or female, aged 18 – 70 years
3. In good general health as evidenced by medical history
4. Peripheral night blood *W. bancrofti* Mf levels >40 Mf/mL
5. No history of taking antifilarial medications in past 12 months
6. Resident of the study area with no plans to change residence in the next 36 months
7. For women of childbearing potential, willing to use appropriate method of contraception for one month following each treatment.

## Exclusion Criteria

An individual who meets any of the following criteria will be excluded from participation in this study:

1. Pregnancy or currently breastfeeding
2. Known allergic reactions to any of the study medications
3. Evidence of severe or systemic comorbidities (aside from features of filarial disease), as judged by the principal investigator.
4. Baseline biochemical abnormalities, as indicated by AST, ALT, or creatinine >2 times the upper limit of normal.
5. Evidence of urinary tract infection as indicated by 3+ nitrites on dipstick (individuals with 1+ or 2+ nitrites will not be excluded) or underlying chronic kidney disease as indicated by 3+ protein or 3+ blood on urine dipstick exam.
6. Hgb <7 gm/dL (any such individuals will be referred to the local health center for evaluation and treatment)
7. Positive skin snip for onchocerciasis

**Justification for exclusion of women and children**

Pregnant and/or breastfeeding women will be excluded because ABZ, IVM, and DEC are contraindicated in pregnancy and there is insufficient data on Mox safety during pregnancy and during breastfeeding. Children will not be included in the trial because Mox is not FDA approved for children aged <12 years, and because it will not be logistically feasible to admit children for inpatient observation in Part 1. Because we will have no safety data for children from Part 1, we will also exclude children from Part 2.

## Screen Failures

Screen failures are defined as participants who consent to participate in the clinical trial but are not subsequently randomly assigned to the study intervention or entered in the study. A minimal set of screen failure information is required to ensure transparent reporting of screen failure participants, to meet the Consolidated Standards of Reporting Trials (CONSORT) publishing requirements and to respond to queries from regulatory authorities. Minimal information includes demography, screen failure details, eligibility criteria, and any SAE.

Individuals who do not meet the criteria for participation in this trial because of W. bancrofti Mf/mL <40 at the time of enrollment, or because of elevated AST, ALT, or Cr, may be rescreened one time. If the rescreening occurs within 30 days of the initial screening, only the test results not meeting inclusion criteria will be repeated at rescreening. If rescreening occurs more than 30 days after initial screening, all screening/baseline tests will be repeated. Rescreened participants will be assigned the same participant number as for the initial screening.

## Strategies for Recruitment and Retention

The study population will be recruited from filariasis-endemic areas of Agboville District (and neighboring districts if necessary) in Côte d’Ivoire. We will attempt to recruit equal numbers of men and women (18 – 70 years of age) who are willing to participate, although the gender balance will be dictated by the availability of subjects meeting inclusion criteria.

**Prescreening**

Potential participants will be identified by the Ministry of Health (MOH), as they will conduct their routine public health assessment of LF transmission in the Agboville District in the summer of 2019. Their activities will include convenience testing of residents in the area for W. bancrofti antigenemia using the Filariasis Test Strip (FTS). In general, the MOH team will coordinate with the village chief and local health officers to publicize the screening. MOH staff will either visit residents at their homes, or, more likely, invite residents to be tested for LF at a pre-arranged location (such as the local dispensary, school, chief’s home, or other common meeting place). Residents with a positive FTS result of 2+ or higher (test line at least as strong as the control line) will be visited at night (between 9:30 PM and 3 AM) to test for W. bancrofti microfilaremia using either thick blood smear (TBS) of a fingerprick blood sample, or Mf filtration of a venous blood sample. This public health assessment will provide the necessary pre-screening information (Mf count) to identify potential participants for the study. Residents found to have W. bancrofti microfilaremia who have not received MDA for LF within the prior 12 months will be eligible for screening for inclusion in the trial.

**Screening**

Individuals meeting prescreening criteria will be contacted by the study team and invited to participate in study screening. We anticipate that screening for Part 1 will be conducted in two parts: 1) a community visit to check nocturnal blood for Mf counts and biochemistries, and urine for dipstick and pregnancy test, and 2) prior to treatment at CRFLA for all other baseline assessments. However, if logistics dictate otherwise, the activities planned for the community visit may be conducted at CRFLA and vice versa. For Part 2, we anticipate that all screening assessments will be done in the community (but may be done at CRFLA if necessary). Screening assessments will include a 10 mL venous blood draw, done at night, for serum biochemistry tests, hemoglobin level, physical exam including vital signs, and Mf counts by filtration of venous blood. All individuals will also be tested for onchocerciasis by examination of skin snip for microfilariae (2 punches, 1 over each iliac crest). Demographic data will be collected. Urine will also be tested by dipstick (all participants) and urine pregnancy test (all female participants) will be performed. Male participants will undergo scrotal ultrasound for identification of filarial worm nests.

**Retention**

Retention in the study will be encouraged/enhanced in several ways. During inpatient observation (Part 1), participants will be housed in a comfortable, air-conditioned ward equipped with a dedicated toilet and television. None of the 66 participants in a prior inpatient study conducted in the same manner and location left the study during the inpatient observation period. Retention during the 3 years of annual follow up (Part 1 and Part 2) will be enhanced by conducting follow-up assessments in participants’ home villages.

**Compensation**

No compensation will be offered for the MOH community LF assessment (pre-screening). Individuals participating in screening for Part 1 or Part 2, and/or the inpatient observation period (Part 1) will be compensated for time and travel expenses (approximately $10/day). No financial compensation will be offered for outpatient follow-up visits. However, at each outpatient follow up visit, participants will receive a care package of nominal value (for example, soap, canned fish, and bread).

# STUDY INTERVENTION

## Study Intervention(s) Administration

### Study Intervention Description

Trial participants will be randomized to receive one of the following interventions:

Arm 1: IVM 200 µg/kg + ABZ 400 mg (**IA**)

Arm 2: Mox 8 mg + ABZ 400 mg (**MoxA**)

Arm 3: IVM 200 µg/kg + DEC 6mg/kg + ABZ 400 mg (**IDA**)

Arm 4: Mox 8 mg + DEC 6mg/kg + ABZ 400 mg (**MoxDA**)

### Dosing and Administration

**Initial treatment**

The study drugs will be taken together orally with liquid. Study drugs will be given as directly observed therapy, and participants will be observed for 30 minutes after swallowing of pills. Dosing will follow the treatment arms as outlined in 6.1.1 above. Treatment in the hospital (Part I) will occur in cohorts of up to 10 participants. This is based on the project’s capacity for transport, examination, and housing of participants. In Part 2, treatment medications will also be administered by study staff and be directly observed, but will occur in the participant’s village.

**Contraception**

Because there is insufficient data about safety of moxidectin during pregnancy, women of childbearing potential must agree to adhere to a reliable method of contraception for a period of one months after each study treatment.

**Repeat treatment**

The IA arm will receive IA annually, which is standard of care for LF MDA in Cote d’Ivoire. All other arms will receive the assigned treatment at the time of enrollment. At the 12 month interim analysis, if IA is found to be superior to MoxA for the primary endpoint, participants in the MoxA arm will be switched to annual IA therapy for the duration of the study. Because it would be unethical to exclude participants from ongoing MDA for the duration of the study, participants in the MoxA, IDA, and MoxDA arms who do not achieve complete clearance of Mf at 24 months will be re-treated with their assigned therapy within one month after the 24 month follow-up visit. All participants who are microfilaremic at the end of the study (after the 36-month assessment) will be offered IA treatment per national LF program guidelines as a bridge back to annual MDA.

## Preparation/Handling/Storage/Accountability

### Acquisition and accountability

Moxidectin will be donated by Medicines Development for Global Health (MDGH). IVM, DEC, and ABZ will be purchased from Merck, GlaxoSmithKline (GSK), and Pfizer (India), respectively.

Medications will be distributed by a study nurse or other designated staff and ingestion directly observed. Unused Moxidectin will be incinerated or returned to MDGH; unused DEC, IVM, and ABZ in the original, sealed, packaging will be donated to the CHR Agboville pharmacy.

### Formulation, Appearance, Packaging, and Labeling

Package inserts for each of the study medications are attached to this protocol as an appendix.

### Product Storage and Stability

Storage and stability requirements for each study drug are listed below. Study medications will be stored at CHR Agboville in a secure, climate-controlled environment *between 18 -25°C*. Site pharmacist will be trained in temperature monitoring for the drugs according to the study SOP for temperature monitoring.

| **Medication** | **Package insert storage recommendations** |
| --- | --- |
| Albendazole (Albenza) | Store between 20° and 25°C |
| Albendazole (Zentel) | Store in a dry place at a temperature not exceeding 30°C |
| DEC | Do not store above 30°C; protect from light and moisture |
| IVM | Store at temperatures below 30°C |
| Moxidectin | Store below 30°C; protect from light |

## Measures to Minimize Bias: Randomization and Blinding

**Blinding**

Recognizing that number and appearance of tablets received in each study arm will be noticeably different, this will be an open label study. To minimize bias, administration of study medications will be performed by a staff member with no role in assessing AEs. AE assessments and all other outcome measures will be assessed by observers masked to the treatment assignment.

**Randomization**

Allocation sequence for arm assignment will be generated by the study statistician prior to study onset. Consenting subjects will be allocated to treatment sequentially according to the randomization list.

## Study Intervention Compliance

Ingestion of study medications will be directly observed.

## Concomitant Therapy

Concomitant medications will be recorded at the time of enrollment/treatment. Medications to be reported in the Case Report Form (CRF) are concomitant prescription medications, over-the-counter medications and supplements taken within 48 hours prior to receiving the assigned study treatment (medial history), and any medications taken during the safety assessment period (days 1-7 post-treatment).

**Antifilarial medications**

Treatment with antifilarial or other anthelmintic medications other than those provided as part of the study is prohibited until after the 36-month assessments are complete. These medications include IVM, DEC, ABZ, mebendazole, levamisole, parantel, and doxycycline. There are no dietary restrictions.

**Other prohibited concomitant medications**

The following medications, listed as having class C interactions with albendazole or ivermectin, will not be permitted at the time of treatment: warfarin (or other vitamin K antagonists), carbamazepine, phenobarbital, phenytoin, azithromycin, erythromycin, or any strong P-glycoprotein inducers/inhibitors, listed in the table below.

| **P-glycoprotein/ABCB1 Inhibitors** | **P-glycoprotein/ABCB1 Inducers** |
| --- | --- |
| Amiodarone, Azithromycin, Carvedilol, Clarithromycin, Cyclosporine, Daclatasvir, Dronedarone, Elagolix, Eliglustat, Erythromycin, Flibanserin, Fostamatinib, Glecaprevir and Pibrentasvir, Itraconazole, Ivacaftor, Ketoconazole, Lapatinib, Ledipasvir, Neratinib, Ombitasvir, Paritaprevir, Propafenone, Quinidine, Quinine, Ranolazine, Ritonavir, Rolapitant, Simeprevir, Velpatasvir, Vemurafenib, Verapamil | Apalutamide, Carbamazepine, Fosphenytoin, Phenytoin, Rifampin, St John's Wort |

# PARTICIPANT DISCONTINUATION/WITHDRAWAL

## Participant Discontinuation/Withdrawal from the Study

Participants are free to withdraw from participation in the study at any time upon request.

An investigator may discontinue or withdraw a participant from the study if any clinical AE, laboratory abnormality, or other medical condition or situation occurs such that continued participation in the study would not be in the best interest of the participant.

The reason for participant discontinuation or withdrawal from the study will be recorded on the Case Report Form (CRF). Subjects who sign the informed consent form and are randomized but do not receive the study intervention may be replaced. Subjects who sign the informed consent form, and are randomized and receive the study intervention, and subsequently withdraw, or are withdrawn or discontinued from the study, will not be replaced.

## Lost to Follow-Up

A participant will be considered lost to follow-up for the primary endpoint if he or she cannot be visited for the 12-month (IA vs MoxA comparison) or 24-month assessment (IDA vs. MoxA comparison) and is unable to be contacted by the study site staff. Persons who miss the 12- or 24-month follow-ups can be tested at subsequent visits.

Before a participant is deemed lost to follow-up, the investigator or designee will make every effort to regain contact with the participant. These contact attempts should be documented in the participant’s medical record or study file.

Should the participant continue to be unreachable, he or she will be considered to have withdrawn from the study with a primary reason of lost to follow-up.

# STUDY ASSESSMENTS AND PROCEDURES

## Efficacy Assessments

The efficacy assessment (procedures) and their timing are summarized in the SOA (section 1.3).

**Stay at the health center (Part 1 only)**

Part 1 calls for collection of multiple blood samples and AE assessments for up to 72 hours after treatment. Because the study subjects may live some distance from the health center and transportation is difficult in the study area, study subjects will be asked to remain in the health center during this time. Part 1 participants will be asked to report at the health center by 6pm. Baseline physical exam, vital signs, AE assessment, and scrotal ultrasound (men only), will be completed the evening of admission. Skin snips will be performed if not done previously. Participants will receive study treatments the following morning and will remain at the health center for 72 hours after treatment. All meals will be provided. CRFLA is equipped with standard hospital beds, private toilets and showers, and appropriate staff support.

**Ultrasound (Part 1 and Part 2)**

Scrotal ultrasound examination will be performed on all men at baseline, and at 6, 12, 24, and 36 months post-treatment to identify the presence and number of adult worm nests. Ultrasound examinations will be done by trained examiners and may be done either at CRFLA or at a private location in the community.

**Treatment**

Study drugs will be administered by study staff as directly observed therapy.

- **Part 1**: Patients will fast overnight, which is consistent with usual eating patterns in Côte d’Ivoire. Following breakfast, study drugs will be administered
- **Part 2**: Treatment medications will be administered in the participant’s village, and the time of day may vary. Because no PK drug levels will be measured in Part 2, there is no requirement for pre-treatment overnight fasting.

**Collection of samples for drug monitoring (Part 1 only)**

For Part 1 samples (5-10 ml) of heparinized blood will be taken pretreatment and at 2, 3, 4, 6, 12, 24, and 48 hours after treatment and at day 7 if there are significant abnormalities at 28 hours. This will be accomplished by inserting an indwelling venous catheter into a vein in the forearm using sterile technique. The needle will remain inserted for the first 12-24 hours after drug administration. Five milliliters of blood will be taken at time points when only drug tests are performed. For the time points when liver function tests, hematological parameters and Mf counts are also to be evaluated, 5 to 10 ml will be removed. Therefore, a total of 70 to 90 ml of blood will be drawn over a period of approximately 1 week that includes the screening of individuals prior to enrollment. Samples will be collected in sterile heparinized tubes. Anticoagulated whole blood will be removed for Mf counts (if needed). The remaining whole blood will be centrifuged. Plasma will be aliquoted into cryotubes in duplicate and frozen (-20°C) for later testing. Efforts must be made to obtain the pharmacokinetic samples at the listed times relative to dosing. However, samples obtained within 10% of the nominal time (e.g., within 6 minutes of a 60 minute sample) from dosing will not be considered as a protocol deviation, as long as the exact time of the sample collection is noted on the source document and CRF.

**Collection of venous blood for MF counts and CFA (Parts 1 and 2)**

Approximately 10 ml of blood will be collected at baseline for screening and Mf counts, and at 6, 12, 24 and 36 months after treatment for Mf counts and CFA assay. An additional 5 ml of blood will be collected on day 7 for participants in Part 1 to assess Mf count.

**Laboratory Tests and Analysis to be performed at local health center in Côte d’Ivoire or other comparable laboratories**

**Diagnosis of LF and Mf counts**

Individuals will be screened for LF infection based on results of the CFA test and by the presence and number of Mf in night blood. Peripheral venous blood will be obtained after 21:00 hours (Mf counts peak at night and are often not detected during the day). Blood will be collected in heparin-coated tubes and 1 mL will be passed through a 5 μM Nuclepore filter. Filters will be dried and stained with Giemsa. The presence and number of Mf on filters will be assessed by light microscopy.

**Blood samples for hematologic and biochemical assays**

Biochemistry tests will be performed on site using a portable Piccolo Biochemistry Analyzer (Abbott Labs). Hemoglobin will be measured with a HemoCue point of care blood test analyzer. Aliquots of plasma collected as described above will be frozen at -20°C. Samples will be stored for up to one month at -20°C until they can be transferred to CSRS for storage at -80°C.

**Urinalysis**

A mid-stream urine will be collected into a clean container. A dipstick that measures a variety of analytes will be used to assess protein, nonhemolyzed blood and nitrates for possible urinary tract infection or chronic kidney disease. Urine samples testing strongly positive (2+ or greater) for nitrates, leukocyte esterase, or blood, will also be centrifuged for microscopic examination for RBC, WBC, and casts.

Samples will be stored for up to one month at -20°C until they can be transferred to CSRS for storage at -80°C until shipment to the United States.

**Skin snips to exclude onchocerciasis**

Two (2) skin snips will be taken (1 from each iliac crest) using a cornea-scleral punch (Holth-type). Each snip will be incubated for at least 30 minutes in isotonic saline in a well of a flat-bottomed microtiter plate. The Mf will be counted using an inverted microscope. The presence and number of *O. volvulus* Mf will be recorded.

**Assays to be performed outside Côte d’Ivoire**

***W. bancrofti* antigen ELISA**

Serum CFA levels will be quantified at Washington University in St. Louis according to the well-established Weil lab protocol (29, 30).

**Plasma drug levels**

Assays for measuring plasma drug levels will be performed in the laboratory of Dr. DJ Murry, College of Pharmacy, University of Nebraska Medical Center, as described below.

*ABZ and metabolites assay*

Plasma concentrations of ABZ, ABZSO, and ABZSO2 will be determined using a validated liquid chromatography-mass spectrometric method using previously published extraction methodology (Kitzman, Cheng et al. 2002). The lower limit of quantification for all analytes was 2 ng/mL and the upper limit of quantitation was: 520 ng/mL for ABZ and ABZSO2; 1040 ng/ mL for ABZSO. The coefficient of variation during validation at LLOQ and the three control levels for intra-day and inter-day precision ranged from 1.6% to 13.8%, and 8.0% to 10.7% for ABZ; 3.9% to 10.1% and 7.3% to 14.6% for ABZSO; and 2.5% to 12.0% and 7.6% to 10.2% for ABZSO2, respectively. The intra-day and inter-day bias ranged from 0.3% to 14.3% and 3.0% to 14.2% for ABZ; -16.2% to 18.5% and -10.6% to 8.8% for ABZSO; and -11.6% to 16.8% and -10.2% to 15.4% for ABZSO2, respectively.

*Diethylcarbamazine assay*

Plasma concentrations of DEC will be determined using a validated liquid chromatography-mass spectrometric method using previously published extraction methodology (Miller and Fleckenstein 2001, Schmidt, King et al. 2014). The calibration curve range is 4 – 2200 ng/mL. The coefficient of variation during validation at the three control levels for intra-day and inter-day precision ranged from 2.1% to 8.2%, and 5.4% to 8.4%, respectively. The intra-day and inter-day bias ranged from -9.7% to 12.7% and 2.2% to 7.5%, respectively.

*Ivermectin assay*

Plasma concentrations of IVM will be determined using a previously published methodology (20). The linear range of the calibration curve is 0.20- 400 ng/mL from 0.20 mL plasma.

*Moxidectin assay*

Plasma concentrations of Mox will be determined using a previously published methodology (31).

**Exploratory assessment of the relationship between cytochrome p450 genotypes and pharmacokinetic parameters**

Individual participant cytochrome p450 genotypes may be determined if the investigators feel such analysis is warranted based on the results of the PK analyses. For these analyses the screening skin snip tissue would be used as a source of genomic DNA (after migration of any *O. volvulus* Mf out of the tissue, the skin snip tissue for participants in Part 1 will be placed in RNAlater and stored at -80°C at CSRS in Cote d’Ivoire). If cytochrome p450 analyses are warranted, samples will be shipped to Washington University or CWRU for analysis. Any unused skin snip samples will be destroyed at study completion. Descriptive statistics for all pharmacokinetic parameters by genotype and drug will be calculated for the genotypes found in more than 3 subjects and jointly displayed. For the genotypes found in 3 or less subjects, the minimum, median and maximum values for all pharmacokinetic parameters by genotype and drug will be displayed. Exploratory plots may be generated if considered useful for presentation of the findings.

**Additional analyses**

The results of the pre-specified analyses may suggest additional *ad hoc* analyses that may be undertaken. These might include, but may not be limited to, analyses of serum/plasma biomarkers (parasite antigens or human cytokines/inflammatory makers/etc), or additional studies of parasite DNA. No human genetic analyses, aside from the cytochrome p450 analysis (if warranted), will be undertaken.

**Specimen Preparation, Handling, and Shipping**

Blood plasma and urine samples will be prepared as described above, aliquoted into cryotubes, labeled using numbered bar codes and frozen at -20°C in a freezer in the laboratory at the clinic where subjects are monitored. Samples will be transferred to a -80°C freezer in Abidjan until shipment to the United States for determination of drug levels. Following the current study, de-identified samples will only be used for other studies if permission was given in the consent form. If not, samples will be destroyed at the completion of the study*.*

Samples will be shipped to the laboratory of Dr. Christopher L. King at Case Western Reserve University, 10900 Euclid Ave, Cleveland, OH 44106, and/or to the laboratory of Dr. Philip Budge at Washington University in St. Louis, 4444 Forest Park Ave, St. Louis, MO 63108. Shipping will following International Air Transport Association guidelines.

## Safety and Other Assessments

Several safeguards are in place to ensure participant safety. No one with evidence of severe comorbidity by history, physical exam, or baseline screening labs will be enrolled in the study. The safety of single dose LF treatment with IDA has been well established. Self-limited AEs are expected following treatment of LF with microfilaricidal medications. As an extra precaution to ensure that AEs after treatment in this trial are consistent with AEs seen in other studies, the first group of at least 48 participants will be monitored closely for AEs at CRFLA for three days following treatment. After at least 12 participants with Mf counts >40 Mf/mL in each group are treated, if no unexpected safety signals are evident, and with approval of the DSMB, the remainder of participants (Part 2) will be treated in the community. Active surveillance for AEs for Part 2 will involve visits from the study team on days 1 and 2 following treatment and passive surveillance by trained village health workers until day 7.

**Monitoring safety variables**

Screening for Cr, AST and ALT will be performed using a portable Piccolo Biochemistry Analyzer (Abbott Labs). Prior to drug administration all subjects will undergo detailed physical examination and urinalysis using a dipstick to detect nitrites, protein, and blood. Immediately before drug administration blood pressure, pulse, and temperature will be taken. Following drug administration, AE assessments including a review of subjective symptoms and vital signs will be performed. If there is any AE with severity grade 2 or greater, or if there are abnormal vital signs, a targeted physical examination will be performed.

During Part 1, AE assessments will be conducted every 6 hours for the first 48 hours then every 12 hours until 72 hours after treatment. If participants are asleep at the AE assessments scheduled for 18 and 42 hours post-treatment, which will occur at approximately 1 AM, study staff may choose to skip the assessment. Urinalysis and venous blood samples to determine serum electrolytes and liver transaminases will be obtained at the time of drug administration, and at 24, 48 and 72 hours after treatment. If abnormal and above baseline levels, these will be repeated on day 7 after treatment.

During Part 2, urinalysis, Cr, AST, and ALT will be collected at the day 2 post-treatment visit. If abnormal and above baseline levels, these will be repeated on day 7 after treatment.

The final study visit for monitoring AEs will be one week following baseline drug administration. Active AE monitoring will not be done following retreatment at 24 months (for those who are Mf positive at 24 months). As per usual MDA protocol, trained village health workers will be available for passive AE surveillance. In addition, study staff will be accessible post-treatment to assess participants who report AEs.

Safety evaluation, AE grading, and safety reporting are described in the accompanying DSMB Charter and Safety Plan (Appendices).

## Adverse Events and Serious Adverse Events

### Definition of Adverse Events (AE)

An AE is any untoward medical occurrence associated with the use of an intervention in humans, whether or not considered intervention-related (21 CFR 312.32 (a)).

### Definition of Serious Adverse Events (SAE)

A SAE is defined as any adverse drug experience occurring at any dose that results in any of the following outcomes:

- death
- life-threatening situation (Study participant is at immediate risk of death)
- inpatient hospitalization or prolongation of existing hospitalization (excluding those for Study therapy or placement of an indwelling catheter, unless associated with other serious events)
- persistent or significant disability/incapacity (substantial disruption of the ability to conduct normal life functions)
- congenital anomaly/birth defect in the offspring of a Study participant who received Study Drug
- Other: Important medical events that may not result in death, be immediately life-threatening, or require hospitalization may be considered serious when, based upon appropriate medical judgment, they may jeopardize the participant and may require medical or surgical intervention to prevent one of the outcomes listed in this definition. Examples of such events are
  - Intensive treatment in an emergency room or at home for allergic bronchospasm
  - Blood dyscrasias or convulsions that do not result in hospitalization
  - Development of drug dependency or drug abuse

For clarification:

- Death is an outcome of an AE and not an AE in itself. In reports of death due to “disease progression”, where no other information is provided, the death will be assumed to have resulted from progression of the disease being treated with the Study Drug(s).
- “Occurring at any dose” does not imply that the participant is receiving Study Drug at the time of the event. Dosing may have been given as treatment cycles or interrupted temporarily prior to the onset of the SAE, but may have contributed to the event.
- “Life-threatening” means that the Study subject was at immediate risk of death from the event as it occurred. This does not include an event that might have led to death, if it had occurred with greater severity.
- Complications that occur during hospitalizations are AEs. If a complication prolongs hospitalization, it is a SAE
- “In-patient hospitalization” means the participant has been formally admitted to a hospital for medical reasons, for any length of time. This may or may not be overnight. It does not include presentation and care within an emergency department.
- The Investigator should attempt to establish a diagnosis of the event based on signs, symptoms and/or other clinical information. In such cases, the diagnosis should be documented as the AE and/or SAE and not the individual signs/symptoms.

### Classification of an Adverse Event

#### Severity of Event

For AEs not included in the protocol defined grading system, the following guidelines will be used to describe severity.

- **Mild (Grade 1)** – Events require minimal or no treatment and do not interfere with the participant’s daily activities such as work or school attendance.
- **Moderate (Grade 2)** –Moderate events may cause some interference with functioning; they prevent the participant from working (at home or at their normal work place) or going to school.
- **Severe (Grade 3)** – Events that interfere with the participant’s usual activities of daily living (eating, dressing, walking in the house, getting out of bed) so that the participant requires assistance from a friend or family member. Of note, the term “severe” does not necessarily equate to “serious”. Persons with severe AEs require medical evaluation to determine whether they have a SAE.
- **Life**-**threatening (Grade 4) -** Requires transfer to medical facility for inpatient hospitalization and treatments. Grade 4 AEs are usually SAEs.
- **Death (Grade 5)**

#### Relationship to Study INTERVENTION

All SAEs must have their relationship to study intervention assessed by the clinician who examines and evaluates the participant based on temporal relationship and his/her clinical judgment. The degree of certainty about causality will be graded using the categories below. In a clinical trial, the study product must always be suspect.

- **Definitely Related** – There is clear evidence to suggest a causal relationship, and other possible contributing factors can be ruled out. The clinical event, including an abnormal laboratory test result, occurs in a plausible time relationship to study intervention administration and cannot be explained by concurrent disease or other drugs or chemicals. The response to withdrawal of the study intervention (de-challenge) should be clinically plausible.
- **Probably Related** – There is evidence to suggest a causal relationship, and the influence of other factors is unlikely. The clinical event, including an abnormal laboratory test result, occurs within a reasonable time after administration of the study intervention, is unlikely to be attributed to concurrent disease or other drugs or chemicals, and follows a clinically reasonable response on withdrawal (de-challenge).
- **Potentially Related** – There is some evidence to suggest a causal relationship (e.g., the event occurred within a reasonable time after administration of the trial medication). However, other factors may have contributed to the event (e.g., the participant’s clinical condition, other concomitant events). Although an AE may rate only as “possibly related” soon after discovery, it can be flagged as requiring more information and later be upgraded to “probably related” or “definitely related”, as appropriate.
- **Unlikely to be related** – A clinical event, including an abnormal laboratory test result, whose temporal relationship to study intervention administration makes a causal relationship improbable (e.g., the event did not occur within a reasonable time after administration of the study intervention) and in which other drugs or chemicals or underlying disease provides plausible explanations (e.g., the participant’s clinical condition, other concomitant treatments).
- **Not Related** – The AE is completely independent of study intervention administration, and/or evidence exists that the event is definitely related to another etiology. There must be an alternative, definitive etiology documented by the clinician.

#### Expectedness

The principal investigators will be responsible for jointly determining whether an AE is expected or unexpected. An AE will be considered unexpected if the nature, severity, or frequency of the event is not consistent with the risk information previously described in the prescribing information (Appendices) for the study medications.

If an AE is graded as severe or serious, the medical monitor will be called upon to give an independent opinion on the expectedness and the relatedness to the DSMB for consideration.

### Time Period and Frequency for Event Assessment and Follow-Up

The occurrence of an AE or SAE may come to the attention of study personnel during study visits or interviews of a study participant presenting for medical care, or upon review by a study monitor.

All AEs including local and systemic reactions not meeting the criteria for SAEs will be captured on the appropriate CRF. Information to be collected includes event description, time of onset, clinician’s assessment of severity, and time of resolution/stabilization of the event. All AEs occurring during the 7-day post-treatment observation period must be documented appropriately regardless of relationship. All AEs will be followed to adequate resolution.

Any medical condition that is present at the time that the participant is screened will be considered as baseline and not reported as an AE. However, if the study participant’s condition deteriorates at any time during the study, it will be recorded as an AE.

Changes in the severity of an AE will be documented as they occur to allow an assessment of the duration of the event at each level of severity to be performed. AEs characterized as intermittent require documentation of onset and duration of each episode.

The site PI will record all reportable events with start dates occurring any time after informed consent is obtained until 7 (for non-serious AEs) or 30 days (for SAEs) after receipt of the most recent study medication. Events will be followed for outcome information until resolution or stabilization.

### Adverse Event Reporting

Procedures for grading and reporting AEs/SAEs during the course of this study are found in the corresponding study Safety Plan (Appendix). In general, AEs will be reported to the DSMB in aggregate at regular intervals for their review and assessment of risk posed to the participants.

### Serious Adverse Event Reporting

Any SAEs occurring after the first does of Study Drug has been administered must be reported.

The study clinician will immediately (within 24 hours of becoming aware of the SAE) report the occurrence of a SAE to the sponsor, whether or not considered study intervention related, including those listed in the Safety Plan. For fatal or life-threatening events, the Investigator is also required to send copies of the available hospital case reports, autopsy reports, and other documents when requested and applicable. A full SAE report form must be submitted according to the Safety Plan procedures including a narrative of the adverse event and an assessment of whether there is a reasonable possibility that the study intervention caused the event. SAEs will be reported to the DSMB, CNER, and Ivorian FDA within 48 hours of the PI becoming aware of the SAE.

All SAEs will be followed until satisfactory resolution or until the site investigator deems the event to be an unavoidable progression of a chronic condition or at such a time as the participant is stabilized. Other supporting documentation of the event may be requested by the Study Sponsor and should be provided as soon as possible.

Sponsor’s reporting to the manufacturer

All SAEs must be reported to the Manufacturer (MDGH) by the Sponsor (Washington University) as soon as is practical and within 24 hours of the Sponsor being notified of the event. Follow up information provided by the Investigator must also be provided to the MDGH within 24 hours of the Sponsor receiving the information. MDGH may request additional information from the Investigator to ensure the timely completion of accurate safety reports.

All deaths, regardless of cause, must be reported to the Company for Study subjects on Study and for deaths occurring within 90 days of last Study Drug dose or within 30 days of last Study evaluation, whichever is longer.

The occurrence and outcome, respectively, of any pregnancy must reported to the Sponsor and the Sponsor must notify MDGH within 48 hours of the Sponsor being notified.

### Reporting of Pregnancy

The Investigator is to report all pregnancies within 48 hours of becoming aware of the pregnancy. Pregnant and breastfeeding women are not eligible for inclusion in the study. However, if a pregnant or breastfeeding woman is accidently treated, the investigators will complete the Pregnancy Report From (included as an Appendix of the study Safety Plan) and submit that to the Washington University IRB, the Ivorian FDA, and the Ivorian CNER within 48 hours of notification. If a pregnant woman is inadvertently dosed, the pregnancy will be followed to its completion (up to one year following the notification) and the investigators will report on the outcome of the pregnancy for mother and fetus/infant using the associated form (also included as an Appendix in the study Safety Plan). Any pregnant woman inadvertently dosed who has a miscarriage or spontaneous abortion will be reported as a SAE. If a participant becomes pregnant more than 30 days after treatment, she may remain in the study, but the study will not actively follow the outcome of the pregnancy.

## Unanticipated Problems

### Definition of Unanticipated Problems (UP)

Unanticipated problems involving risks to participants or others to include, in general, any incident, experience, or outcome that meets all of the following criteria:

- Unexpected in terms of nature, severity, or frequency given (a) the research procedures that are described in the protocol-related documents, such as the Institutional Review Board (IRB)-approved research protocol and informed consent document; and (b) the characteristics of the participant population being studied;
- Related or possibly related to participation in the research (“possibly related” means there is a reasonable possibility that the incident, experience, or outcome may have been caused by the procedures involved in the research); and
- Suggests that the research places participants or others at a greater risk of harm (including physical, psychological, economic, or social harm) than was previously known or recognized.

### Unanticipated Problem Reporting

The site investigator in collaboration with the off-site lead PI will report UPs to the reviewing IRB, the Ivorian CNER, and the Ivorian FDA within 48 hours of becoming aware of the UP. The UP report will include the following information:

- Protocol identifying information: protocol title and number, PI’s name, and the IRB project number;
- A detailed description of the event, incident, experience, or outcome;
- An explanation of the basis for determining that the event, incident, experience, or outcome represents an UP;
- A description of any changes to the protocol or other corrective actions that have been taken or are proposed in response to the UP.

To satisfy the requirement for prompt reporting, UPs will be reported using the following timeline:

- UPs that are SAEs will be reported
  - to the off-site investigators / sponsor within 24 hours (1 calendar day) of the site investigator becoming aware of the event.
  - to the Ivorian FDA and Ivorian CNER within 48 hours (2 calendar days) of the investigator becoming aware of the event.
  - to Washington University IRB within 7 calendar days of the investigator becoming aware of the event
- Any other UP will be reported to the study sponsor within two weeks of the investigator becoming aware of the problem. These will be reported to the Ivorian ERC and FDA and to the Washington University IRB within two weeks.

# STATISTICAL CONSIDERATIONS

## Statistical Hypotheses

The proposed study is a four-armed (IA annually, MoxA, IDA, and MoxDA), randomized, observer-masked study to determine the efficacy of moxidectin combination therapy for treating LF in an MDA setting.

**Hypotheses to be tested**:

1. MoxA is **superior** to IA for clearance of *W. bancrofti* Mf at 12 months post-treatment
2. MoxDA is **superior** to IDA for clearance of *W. bancrofti* Mf at 24 months post-treatment

**Primary Efficacy Endpoint(s):**

- The proportion of each study arm experiencing complete clearance of *W. bancrofti* Mf at 12 (IA vs. MoxA comparison) or 24 months (IDA vs. MoxDA comparison) after treatment

**Secondary Efficacy Endpoint(s):**

- The proportion of participants in each study arm with complete clearance of *W. bancrofti* microfilaremia at 6, 12, 24, and 36 months after treatment
- Reduction in Mf counts (relative to baseline) at 6, 12, and 24 months
- Reduction in CFA levels (relative to baseline) at 6, 12, and 24 months
- Percent inactivation of worm nests as assessed by ultrasound at 6, 12, and 24 months after treatment. Reduction in worm nest count (number of worm nests in all male participants, not just among those with nests at baseline) at the same time points will also be assessed.

An exploratory analysis of each of the stated endpoints at 36 months is also anticipated; the utility of this analysis will depend on how many participants in the MoxA, IDA, and MoxDA groups require retreatment at 24 months.

## Sample Size Determination

A prior clinical trial in Cote d’Ivoire found that IDA achieved prolonged clearance of Mf in about 50% of participants at 24 months. This study is powered to test the hypothesis that MoxDA is superior to IDA for achieving prolonged Mf clearance (80% after MoxDA compared to 50% after IDA at 24 months). A sample size of 39 per group is required to have 80% power to detect such a difference in proportions with 2-sided type 1 error rate of 0.05. Assuming a loss to follow up rate of up to 22%, we will need to enroll 50 participants per arm (200 total) to be able to detect the anticipated difference.

A prior clinical trial in Cote d’Ivoire found that IA achieved complete clearance of Mf in 26% of participants at 12 months. A sample size of 44 per group will have 80% power to show a statistically significant difference between MoxA and IA, if MoxA achieves clearance in 55% of participants at 12 months (compared to 26% for IA). Anticipating a loss to follow-up rate of 12% at 12 months, enrollment of 50 participants will also provide an adequate sample size for this comparison.

Although 50 participants per arm will be necessary to test the primary outcomes at 80% power, we aim to enroll additional participants, up to 62 per group (248 total), if feasible. This will allow better evaluation of secondary outcomes and protect against worse-than-anticipated loss to follow-up.

## Populations for Analyses

A modified intention-to-treat (ITT) analytic approach will be conducted that includes all participants that were randomized and have non-missing outcomes at any assessment. Sensitivity analyses may be conducted to evaluate the appropriateness of the assumptions made in our analyses. These may include multiple imputation, setting missing outcomes to failure to clear Mf, and setting missing outcomes to complete clearance of Mf.

An interim safety analysis will be performed following treatment of the first group participants (Part 1) to ensure that the frequency, nature, and severity of AEs occurring after receipt of Mox-combination therapy are consistent with AEs following IVM-combination therapy, and with historical data. No pre-specified interim efficacy analyses are anticipated, but the primary efficacy endpoint for the IA vs. MoxA comparison is at 12 months and will determine whether the MoxA arm is continued to study duration or is switched to annual IA treatment.

## Statistical Analyses

### General Approach

For descriptive statistics, categorical data will be presented as proportions (or percentages) and continuous data will be presented as means (or geometric means) with standard deviations if normally distributed or as median, range, and interquartile range if non-normally distributed. For the Mf clearance outcome, the subset of participants that are Mf positive in the MoxA, IDA, and MoxDA arms will be retreated. In our analysis of Mf clearance, these retreated subjects (those Mf positive at 24 months) will be assumed to be Mf positive at 36 months (i.e. the 24-month clearance outcome for Mf positive subjects will be carried forward to 36 months). For inferential tests a two-sided p-value of 0.05 will be used to define statistical significance.

Covariates may be pre-specified below or may be added later in the statistical analysis plan (SAP).

### Analysis of the Primary Efficacy Endpoint(s)

For the primary endpoints (clearance of Mf at 12 or 24 months), results will be presented as odds ratios (with confidence bounds) and the proportion of participants in each arm that achieve complete clearance. A repeated measures, logistic regression analysis will be used to assess differences in proportion of participants achieving complete Mf clearance between MoxDA and IDA, and between MoxA and IA. This analysis will adjust for non-independence due to multiple measurements on the same individual. We will also consider adjustment for covariates such as age, sex, and baseline Mf count. The primary endpoints will be assessed using planned comparisons to test the statistical null hypotheses of (1) no difference in the proportion of participants achieving complete Mf clearance between MoxA and IA at 12 months, and (2) no difference in the proportion of participants achieving complete Mf clearance between MoxDA and IDA at 24 months. If the Mox treatments have a higher proportion achieving complete Mf clearance than their corresponding IVM treatments, then we will conclude that the Mox treatments are superior. This same statistical model will also be used for analysis of Mf clearance secondary endpoints.

In the unlikely event that we find that IA has statistically greater clearance than MoxA at 12 months, the MoxA treatment group will be administered IA annually for the remainder of the study. In this case, each of the four groups will still be treated as separate groups in the statistical analyses.

### Analysis of the Secondary Endpoint(s)

**Mf clearance at 6, 12, 24, and 36 months** will be reported as odds ratios with confidence bounds and the proportion of participants that have complete clearance at these time points. Because these endpoints are contained within the same statistical model as the primary endpoints (clearance at 12 and 24 months), we will use the same analytical approach to compare differences in proportions between treatments.

**Reduction in Mf count (6, 12, and 24 months)** will be reported as the reduction in Mf count compared to baseline. Treatment arms will be compared using a repeated measures analysis to account for non-independence of the outcomes due to multiple measurements on each participant. We will consider adjustment for covariates such as age, sex, and baseline Mf count. If the data are highly non-normal then an appropriate data transformation (e.g. logarithmic) or a statistical model that assumes a different distribution (e.g. negative binomial) will be used. We will also compare Mf “area under the curve” (baseline – 24 months) among treatment groups to generate an estimate of transmission potential (number of Mf/mL in blood over time). In addition to the change in Mf count, percentage change in Mf count (relative to baseline) will be analyzed. If these results are appropriately distributed and provide similar statistical inferences as the change in Mf count, then estimates of percentage change will also be reported to aid in clinical interpretation of the results.

**Reduction in circulating filarial antigen** **(6, 12, and 24 months)** compared to baseline will be analyzed in the same manner as reduction in Mf count.

**Presence and number of adult worm nests** will be assessed for all men at baseline and each follow up visit (6, 12, and 24months). The primary focus for this endpoint will be at 12 months post-treatment. Any macrofilaricidal effects of treatment should be evident by 12 months, while the presence of worm nests at 24 or 36 months might represent re-infection rather than persistence. Because *reduction* in worm nests can only be assessed for the subset of male patients that have active worm nests at baseline, one or both of the following analyses will be performed, depending on the proportion of participants found to have nests at baseline and the number of worm nests per individual.

1. **Percent inactivation of adult worm nests.** The proportion of participants in each group having worm nests at baseline who no longer have worm nests. This will be a binary analysis similar to the primary outcome (complete clearance of Mf counts).
2. **Reduction in worm nest counts.** This will be analyzed as reduction in number of worm nests from baseline. For each participant, the number of nests present at follow-up will be subtracted from baseline and the difference (both as a percentage of the baseline, and as a count) will be compared between groups. This will be a similar analysis to the Mf and circulating filarial antigen analyses.

The proportion of male participants without worm nests at baseline who are found to have worm nests at 6, 12, 24, and 36 months will also be reported, and may indicate the risk of re-infection and/or the sensitivity of the assay.

**Pharmacokinetic analyses**

This sub-study will test the hypothesis that Mox has no effect on the PK parameters of DEC and ABZ (including ABZSO).

Noncompartmental pharmacokinetic analyses of DEC, ABZ, ABZSO, ABZSO2, IVM and Mox concentrations will be conducted using WinNonlin (Pharsight Corporation; Cary, North Carolina, USA). All calculations will use the nominal blood sampling times as actual times should actual times be unavailable. The following parameters will be estimated for the five analytes:

- **Cmax:** Peak observed concentration post-dose.
- **Tmax:** Time corresponding to Cmax.
- **Half-life:** computed as ln(2)/Kel, with Kel being magnitude of the slope of the linear regression of the log concentration vs. time profile during the terminal phase. Where R2adj is <0.85 this parameter will be reported as NE (not estimated).
- **AUC0-t:** Area under the concentration-time curve from Hour 0 through LQCT, where LQCT is the time at which the last quantifiable concentration was obtained. Where R2adj is <0.85 this parameter will be reported as NE (not estimated). For analytes that did not have at least eight calculated values, means and the standard deviation would not be calculated.
- **AUC0-inf:** Area under the concentration-time curve from 0 to infinity, computed using AUC0-t + CLQCT / Kel where CLQCT is the last quantifiable concentration at LQCT. Where R2adj is <0.85 this parameter will be reported as NE (not estimated). For analytes that did not have at least eight calculated values, means and the standard deviation would not be calculated.
- **CL/F:** Apparent Clearance, will be computed as Dose/AUC0-inf; for ABZ metabolites, dose will be adjusted for molecular weight differences relative to the parent. Where R2adj is <0.85 this parameter will be reported as NE (not estimated).
- **Vz/F:** Apparent Volume of Distribution, computed as Dose/(Kel*AUC0-inf); for ABZ metabolites, Dose will be adjusted for molecular weight differences relative to the parent. Where R2adj is <0.85 this parameter was reported as NE (not estimated).

For all analytes, AUC parameters will be estimated using a linear-log trapezoidal method. The pharmacokinetic parameter estimates obtained for each of the analytes will be presented by treatment group with provided descriptive statistics including arithmetic mean, standard deviation, coefficient of variation, geometric mean, median, minimum, and maximum. Separate descriptive statistics will be evaluated by gender. For all analytes, exposure parameters (Cmax, AUC0-t, and AUC0-inf) adjusted for mg/kg dose are provided. For ivermectin, parameter estimates will be adjusted to 200 mcg/kg. For DEC, parameters are adjusted to 6 mg/kg. For ABZ, ABZSO, ABZSO2, and Mox, parameters are adjusted to a common mg/kg albendazole or Mox dose.

For the drug-disease interaction analysis, individual DEC, ABZSO, and ABZSO2 exposure parameters (Cmax, AUC0-t, AUC0-inf) will be natural log transformed. Then, for each analyte and parameter, a 90% confidence interval was constructed, in accordance with an independent samples t-test for the difference in means between the four study regimens for the Ln transformed parameters. The point estimate and limits of each 90% CI will be then exponentiated to yield a 90% CI for the ratio of geometric means for each parameter/analyte combination. Comparison of pharmacokinetic parameters between treatments will be assessed by ANOVA with treatment as fixed effect and subject within treatment as random effect. Exploratory analyses with microfilaria count as a categorical or continuous variable may be considered.

**Analysis of plasma drug concentrations**

Drug plasma concentrations and computed pharmacokinetic parameters will be listed by subject and summarized by drug or metabolite (geometric mean with coefficient of variation, arithmetic mean with standard deviation, minimum, maximum, number of observations). Individual and geometric mean (by time) concentrations versus time will be plotted for each treatment group on both linear and natural logarithm scales.

**Assessment of pharmacokinetic parameters**

The geometric mean, median, arithmetic mean, standard deviation, geometric coefficient of variation, minimum, and maximum values for the pharmacokinetic parameters [AUC0-t (primary evaluation parameter), AUC0-inf, t1/2, Tmax, Cmax, and residual area of DEC, ABZ, ABZSO, Mox and IVM will be reported. Exploratory analyses may be conducted as indicated by the data.

### Safety Analyses

We will test the hypothesis that the type and frequency of AEs grade >2 will be the same whether Mox combination therapy or IVM combination therapy is given. AEs will be MedRA-coded and analyzed both as the proportion of participants experiencing each AE (or AE category), and the proportion of participants experiencing any AE. SAEs will be presented in a table or listing.

### Baseline Descriptive Statistics

Intervention groups will be compared on baseline characteristics, including demographics (age and gender) and laboratory/ultrasound measurements, using descriptive statistics.

### Planned Interim Analyses

One interim analysis for safety will be conducted after Part 1 (inpatient/pharmacokinetics) has been completed. The primary outcome for this analysis will be proportion of participants experiencing AEs. These data will be reviewed by the DSMB for the purpose of determining whether the number and severity of AEs are consistent with AEs expected following single-dose treatment (27). The DSMB will then make a recommendation about moving forward with treatment in the community (Part 2). Because the number of treated participants at the time of this analysis will be small, the decision to proceed to community-based treatment or to continue inpatient treatment/monitoring will not rely on inferential tests of hypothesis.

Additional interim analyses will be performed at 6, 12, and 36 months post-treatment to evaluate secondary endpoints.

### Sub-Group Analyses

The pharmacokinetic parameter will be analyzed separately by sex, as well as in aggregate. Other endpoints may be analyzed by subgroups, but no other subgroups are pre-specified, given the small sample size.

# SUPPORTING DOCUMENTATION AND OPERATIONAL CONSIDERATIONS

## Regulatory, Ethical, and Study Oversight Considerations

### Informed Consent Process

#### Consent/assent and Other Informational Documents Provided to participants

Consent forms describing in detail the study intervention, study procedures, and risks are given to the participant and written documentation of informed consent is required prior to screening or administering study drugs. The following consent materials are submitted with this protocol.

- Informed consent for study enrollment English (Appendix)
- Informed consent for study enrollment, French (Appendix)

#### Consent Procedures and Documentation

**Description of the informed consent process**

The informed consent process will start before enrolment of participants, and it will involve both community and individual consent. The informed consent process recognizes the community and cultural values in Côte d’Ivoire and in Agboville district where the study participants reside. Extensive discussion of risks and possible benefits of participation in this study will be provided to the community, the study participants and their relatives. This will be accomplished through a series of community meetings in which the investigators, co-investigators and Ministry of Health representatives explain and discuss the purposes of the research study to residents at study sites and to field assistants who may refer study participants for more information about this study. The investigators and study personnel who will obtain consent from study participants all have received training in human subjects’ regulations and good clinical practice (GCP).

Following consent by community leaders to include participants of their village into the study, the study team will invite all interested adult community members in areas known to have levels of LF infection to attend information meetings held in several central locations throughout the study areas. At these meetings, the study team will describe the purpose and significance of the study, the procedures to be followed, the risks and benefits of participation, and state that participation in the study is voluntary and that declining to participate will not reduce the level of, or access to, health care for the eligible individuals.

Consent forms describing in detail the study procedures and risks will be read at information sessions in French. Formal, written informed consent will be obtained for individuals willing to participate in the study and on the evening prior to admission into the health clinic. The subject will be asked to read, or have read to them, and review the informed consent documents. Upon reviewing the document, the investigator and/or study staff will explain the research study to the subject and answer any questions that may arise.

Only the principal investigators or study staff authorized and certified to obtain informed consent will consent subjects for this study. Only individuals who have signed the consent form and meet eligibility criteria will be enrolled in the study.

Entry into the study and participation will be strictly voluntary. It will be made clear that refusal to participate or a decision to withdraw can occur at any time throughout the course of the study and will not influence their rights or the care they receive at local health facilities. Potential participants will be told that all of their health information will be confidential and that records will be coded without personal identifiers before they are shared with statisticians or project scientists outside of Côte d’Ivoire. They will also be told that no monetary or other gains are offered in exchange for participation apart from compensation for time and reimbursement of travel expenses as described above.

**Comprehension of informed consent**

Potential participants will have an opportunity to ask additional questions after the information session and when they are being consented. To assess comprehension of informed consent and the study, the principal investigator and/or authorized consent study staff will ask the following questions:

1. Do you understand the consent form?
2. Do you have to participate in this study?
3. Will you stay overnight at the Health Center during the study? (Part 1 only)
4. Will we take blood from you during this study?
5. Can you refuse to participate in the study at any time?
6. Is there any charge for being in the study?
7. Will you receive any money for being in the study?
8. Do you know who to call if you have questions?

The responses will be documented on the consent form, signed by the research staff or principal investigator, and a copy given to the study volunteer. In the event a subject indicates a lack of understanding of the study, or any aspect of it, the Principal Investigator/study staff will invite questions and offer explanations of any particular point. If, in the judgment of the Principal Investigator, the subject’s response still does not reflect an understanding of the study, the subject will not be enrolled in the study.

**Provisions for Subjects from Vulnerable Populations**

No subjects from vulnerable populations will be included. Only adult subjects that are otherwise healthy and fully capable of making independent decisions on their own will be included.

**Plan for women who are pregnant while on study**

Women who are pregnant or breast feeding will not be eligible to enroll in this study because of the unknown effects of the drugs and drug combination used in this study. Women of child bearing age who wish to enroll in the study will have a pregnancy test prior to enrolment in the study to ensure they are not pregnant. If they are pregnant, they will be eligible for routine MDA for LF that is provided by the Ministry of Health after they deliver.

**Plan for Inclusion of Illiterate Subjects**

Study participants, if illiterate, will have the information sheet or consent form read to them in the local language by a trained field assistant. Their signature or mark will be witnessed on the consent form, by a literate person who understands French and is independent of the study. For illiterate people who do not know French, the field assistant should use the form to explain the study to the participant in his/her local language.

### Study Discontinuation and Closure

This study may be temporarily suspended or prematurely terminated by the principal investigators if there is sufficient reasonable cause. The suspending or terminating party will provide written notification, documenting the reason for study suspension or termination, to study participants, investigators, and the funding agency. If the study is prematurely terminated or suspended, the Principal Investigator (PI) will promptly inform study participants, the CNER, IRB, the DSMB, and sponsor and will provide the reason(s) for the termination or suspension. Study participants will be contacted, as applicable, and be informed of changes to study visit schedule.

Circumstances that may warrant termination or suspension include, but are not limited to:

- Determination of unexpected, significant, or unacceptable risk to participants. A single treatment-associated SAE is sufficient to suspend the study, since no SAEs are expected.
- Data that are not sufficiently complete and/or evaluable

If suspended, the study may resume once concerns about safety, protocol compliance, and data quality are addressed, and satisfy the sponsor, IRB, DSMB and/or CNER.

### Confidentiality and Privacy

Privacy of the study participants will be maintained by assigning study participants a unique study identification number (UNID). All data, blood samples and laboratory results will be recorded and analyzed by UNID with no personal identifiers. All information collected, including demographic information about enrolled subjects will be kept confidential and available only to the investigators and authorized study personnel such as the data manager. After the public information sessions, potential participants in the study will have an opportunity to sign the consent form in a private place if they choose to be part of the study.

All written forms (i.e. consent and data collection forms) will be securely stored at CRLFA, or alternative secured location. All forms will be labeled and filed in a metal, locked filing cabinet in a secure room with a locking door. Keys will be kept by the Data Manager assigned to the study. All data will be entered into a secure electronic data capture system by trained study staff. The electronic database will be password protected and access to password will be authorized by the Principal Investigators. Electronic data files will be stored on HIPAA-compliant Washington University (or alternative group-operated and –dedicated) server. The paper forms will be stored for the duration of the study plus three years. The electronic database will be stored indefinitely by the PI’s.

The Principal Investigators, Co-investigators and key personnel may use the results of this study for publications, presentations at scientific meetings or as preliminary data for subsequent grant applications. Confidentiality of study participants will be maintained by not including names or personal identifiers in records transferred outside of Cote d’Ivoire. Identifiers will be included in a participant key that will stay in Cote d’Ivoire.

The study site Project Coordinator will permit access to all documents and records that may require inspection by the funding agencies, governmental regulatory agencies, institutional review boards (both Washington University IRB and CNER) or its authorized representatives.

The informed consent process and forms are provided in more detail in the appendices.

### Future Use of Stored Specimens and Data

Data collected for this study will be analyzed and stored at Washington University in St. Louis. After the study is completed, the de-identified, archived data will be transmitted to and stored at the Digital Research Materials Repository at Washington University in St. Louis for use by other researchers including those outside of the study.

With the participant’s approval and as approved by local IRBs, de-identified biological samples will be stored at Washington University in St. Louis, or at Case Western Reserve University with the same goal as the sharing of data. These samples may be used to study the causes of LF, biomarkers for infections, complications of filarial infection or treatment, and other conditions for which individuals with LF are at increased risk, and to improve treatment. Stored samples will also be provided with a code-link that will allow linking the biological specimens with the phenotypic data from each participant, maintaining the blinding of the identity of the participant.

During the conduct of the study, an individual participant can choose to withdraw consent to have biological specimens stored for future research. However, withdrawal of consent with regard to biosample storage may not be possible after the study is completed.

When the study is completed, access to study data will be provided through Washington University in St. Louis. Study samples will not be shared with scientists or institutions that are not participants in the study.

### Key Roles and Study Governance

| **Principal Investigators** | **Medical Monitor** |
| --- | --- |
| GABO Toki Pascal, MD (Site PI) | COFFIE Justin Patrick |
| Centre Hospitalier Regional d’Agboville, Côte d’Ivoire | PAC-CI, Programme Franco-Ivoirian De Recherche Sur Le Vih/Sida Et Les Maladies Associées De Côte D’ivoire. |
| 225 57 45 96 91 | 18+225 02 02 47 66 / +225 21 75 59 60 |
| gabotokipascal@gmail.com | Emailahuacthi@gmail.com |
|  |  |
|  | **Co-Investigators** |
| KOUDOU Benjamin | WEIL Gary |
| BJERUM Catherine | FISCHER Peter |
| BUDGE Philip | KING Christopher |

The principal investigators ultimately will be responsible for the ethical conduct and compliance of the trial. The PIs and co-investigators listed on the title page comprise a Steering Committee responsible for overseeing safety and operation of the study.

A local Study Monitor will be hired on a contract basis to ensure the logistical and ethical compliance of the trial per ICH standards and Ivorian standards. This will include a pre-study GCP training, site assessment and site initiation monitoring. The study will also contract a Medical Monitor, a physician, who will serve to review and evaluate SAEs in collaboration with the site PI of the study. This independent clinician will be a local Ivorian. Duties will be to provide un-biased opinion on the narrative of the SAE given review of the CRF and relevant diagnostic exam results. The site PI and the U.S. PIs will have regular teleconferences to collaboratively reach decisions regarding the conduct and management decisions of the trial.

In support of the investigators there are two project management teams – a site team and a U.S. team. The U.S team is comprised of a project manager and a data manager who directly support the execution and management of the study under the direction of the U.S. PIs. This team provides training and technical assistance to the site team. The site team is comprised of physicians, local data managers, nurses, technicians, laboratory staff, and other staff as needed. In the day to day conduct of the study, the site team reports directly to the site PI. Delegation logs will be maintained throughout the trial to document responsibilities.

### Safety Oversight

Safety oversight will be under the direction of a Data and Safety Monitoring Board (DSMB) composed of individuals with the appropriate expertise, including at least one member with subject matter expertise in LF, one clinical statistician, and one Ivorian physician or scientist. Members of the DSMB will be independent from the study conduct and free of conflicts of interest. The DSMB will oversee project safety and will therefore be active only during the treatment phase of the study. The DMSB will operate under the rules of an approved charter that will be written and reviewed at the organizational meeting of the DSMB, at which time each data element that the DSMB needs to assess will be clearly defined. The DSMB will provide its input to the study investigators and study. The DSMB will meet before the first participant is treated to approve a charter and discuss the protocol. At a minimum, the DSMB will also meet after the inpatient study (Part 1) is completed to review the safety results, and if any SAE is reported (treatment-related or not). It is anticipated that DMSB work will end one week after the last participant receives his/her baseline study medications. The DSMB will not be asked to assess the safety of re-treatment of those who are Mf positive at 24 months, since re-treatment will be done according to standard MOH MDA practices.

### Clinical Monitoring

Clinical site monitoring is conducted to ensure that the rights and well-being of trial participants are protected, that the reported trial data are accurate, complete, and verifiable, and that the conduct of the trial is in compliance with the currently approved protocol/amendment(s), with ICH GCP, and with applicable regulatory requirements.

Study monitoring will be the responsibility of a qualified clinical monitoring group acceptable to CNER. Upon successful approval of the protocol and establishment of the regulatory file, the clinical monitor will establish a clinical monitoring plan. To ensure that the investigator and the study staff understand and accept their defined responsibilities, the clinical monitor will maintain regular correspondence with the site and may be present during the course of the study to verify the acceptability of the facilities, compliance with the investigational plan and relevant regulations, and the maintenance of complete records. As needed, the clinical monitor may witness the informed consent process or other applicable study procedures to assure the safety of subjects and the investigators’ compliance with the protocol and GCP.

### Quality Assurance and Quality Control

Quality control (QC) procedures will be implemented beginning with the data entry system. Edit checks will be programmed into the electronic data capture (EDC) system with auditable logs. Data QC checks that will be run on the database will be generated on a regular basis. Any missing data or data anomalies will be communicated to the site(s) for query clarification/resolution, preferably during the time while the study teams are in the catchment areas.

Following written Standard Operating Procedures (SOPs), the monitors will verify that the clinical trial is conducted and data are generated and biological specimens are collected, documented (recorded), and reported in compliance with the protocol, ICH GCP, and applicable regulatory requirements.

The investigational site will provide direct access to all trial related sites, source data/documents, and reports for the purpose of monitoring and auditing by the sponsor, and inspection by local and regulatory authorities.

### Data Handling and Record Keeping

#### Data Collection and Management Responsibilities

CRFs and SOPs have been developed and will be further updated. All data will be either directly entered into an EDC system on tablets (clinical data) or entered on printed forms (laboratory and ultrasound data) and subsequently entered into the database. Data management will be performed by persons specifically assigned to monitoring and supervising the data entry, its veracity, and confidentiality. Data from the EDC systems will be synced to a secured and dedicated cloud server on a regular basis by the on-site data manager. Final database will be stored and archived at the institutions of the study PIs. Data will only be shared with personnel with data sharing agreements and will be de-identified. All written records will be maintained in locked filing cabinets in a designated room in the originating country and will be stored as long as the local in-country IRB requires.

**Data Capture Methods**

Data will be electronically entered into the EDC system on portable wifi-enabled tablets by study personnel who will be trained in its use prior to enrolment. Paper CRFs will be used as emergency back ups and entered into the EDC systems as soon as possible.

**Types of Data Collected**

This study will collect the following types of data on each study participant. The only information that will be recorded in the subject’s permanent medical record is that they were diagnosed with LF and received a single dose of treatment.

- Demographic data (at enrolment only). This will include age, sex, and village of residence
- Medical history and examination: General physical examination that will include BP, pulse, and temperature both at the beginning of the study and throughout the treatment period. Prior history of any symptoms related to LF; chronic symptoms and signs of lymphedema and/or hydroceles and history of acute symptoms of lymphangitis and lymphadenitis at onset of the study.
- Treatment data – although no treatment for LF will have been given to the study populations within the prior 12 months, other drugs subjects might be taking or have recently taken will be recorded.
- Subjective behavioral and clinical data (e.g. feeling of nausea, neurological or other subjective data related to possible side effects). Such evaluations will be performed at baseline prior to treatment and following treatment.
- Clinical laboratory results, electrolytes and biochemical data
- Prevalence and intensity of Mf levels
- Participant height and weight
- Drug levels (Mox, IVM, ABZ, DEC)
- Results of ultrasound examinations for detection of adult worm nests

#### Study Records Retention

Study documents will be retained for a minimum of 7 years after the last study visit is complete. Documents will be retained longer if required by local regulations. No records will be destroyed without the written consent of the sponsor. It is the responsibility of the sponsor to inform the investigator when these documents no longer need to be retained.

### Protocol Deviations

A protocol deviation is any noncompliance with the clinical trial protocol, ICH GCP, or Manual of Procedures (MOP) requirements. The noncompliance may be either on the part of the participant, the investigator, or the study site staff. As a result of deviations, corrective actions are to be developed by the site and implemented promptly.

It is the responsibility of the site investigator to use continuous vigilance to identify and report deviations within 5 working days of identification of the protocol deviation. All deviations must be addressed in study source documents. Protocol deviations that affect the safety of subjects and/or the integrity of the study must be sent to the reviewing Ivorian CNER and Washington University IRB per their policies. The site investigator is responsible for knowing and adhering to the reviewing IRB requirements. Further details about the handling of protocol deviations will be included in the MOP.

### Publication and Data Sharing Policy

Data will be owned by CSRS. However, it is understood that CSRS will share study data with PIs, co-investigators, and key personnel for use in publications, presentations at scientific meetings or as preliminary data for subsequent grant applications. Confidentiality of study participants will be maintained by not using names or personal identifiers in both verbal and electronic communications between the site PI and the US PIs. The project will be subject to the DOLF Project Data Sharing and Publication Policy (Appendix).

CSRS will permit access to all documents and records that may require inspection by the funding agencies, the Ivorian FDA, CNER, the Washington University IRB, or its authorized representatives.

**Publication and Authorship**

The results of the study and any interim analyses will be published in one or more papers with authorship from the Cote d’Ivoire and the DOLF team. Authorship will be restricted to those persons that had significant input into the design, implementation, analysis of the study data, plus significant participation in writing or editing the manuscript. A committee comprising the PIs and co-investigators listed on the title page will determine when, where and with what authorship order the data will be published.

## Abbreviations

| ABZ | Albendazole |
| --- | --- |
| ABZSO | Albendazole Sulfoxide |
| AE | Adverse Event |
| ALT | Alanine Aminotransferase |
| ANOVA | Analysis of Variance |
| AST | Aspartate Aminotransferase |
| AUC | Area Under the Curve |
| CHR | Centre Hospitalier Regionale (Regional Central Hospital) |
| CLQCT | Concentration at Last Quantifiable Concentration Time |
| CONSORT | Consolidated Standards of Reporting Trials |
| Cr | Creatinine |
| CRF | Case Report Form |
| CRFLA | Centre de Recherche de Filariose Lymphatique d’Agboville (Filariasis Research Center, Agboville) |
| CSRS | Centre Suisse de Recherches Scientifiques (Swiss Scientific Research Center) |
| CYP3A4 | Cytochrome P450-3A4 |
| DEC | Diethylcarbamazine |
| DOLF | Death to Onchocerciasis and Lymphatic Filariasis Project |
| DOT | Directly Observed Treatment |
| DSMB | Data Safety Monitoring Board |
| eCRF | Electronic Case Report Form |
| ELISA | Enzyme-Linked-Immunosorbent Assay |
| ERC | Ethics Review Committee |
| FA | Filarial Antigen |
| FDA | Food and Drug Administration |
| FTS | Filariasis Test Strip |
| GABA | Gamma-Aminobutyric Acid |
| GCP | Good Clinical Practices |
| GPELF | Global Programme to Eliminate Lymphatic Filariasis |
| GSK | GlaxoSmithKline |
| Hgb | Hemoglobin |
| IA | Ivermectin/Albendazole |
| ICH GCP | International Conference on Harmonization of Good Clinical Practice |
| IDA | Ivermectin/DEC/Albendazole |
| IRB | Institutional Review Board |
| ITT | Intention-to-Treat |
| IVM | Ivermectin |
| LF | Lymphatic Filariasis |
| LQCT | Last Quantifiable Concentration Time |
| MDA | Mass Drug Administration |
| MDGH | Medicines Development for Global Health |
| MedDRA | Medical Dictionary for Regulatory Activities |
| Mf | Microfilaria |
| MOH | Ministry of Health |
| MOP | Manual of Procedures |
| Mox | Moxidectin |
| MoxA | Moxidectin/Albendazole |
| MoxDA | Moxidectin/DEC/Albendazole |
| NCT | National Clinical Trial |
| PI | Principal Investigator |
| PK | Pharmacokinetics |
| PNL-SGF | Programme National de la Lutte Contre la Schistosomiase, les Geohelminthiases et la Filariose Lymphatique |
| QC | Quality Control |
| SAE | Serious Adverse Event |
| SAP | Statistical Analysis Plan |
| SOA | Schedule of Activities |
| SS | Skin Snip |
| STH | Soil-Transmitted Helminths |
| TBS | Thick Blood Smear |
| U/S | Ultrasound |
| UA | Urinalysis |
| UP | Unanticipated Problem |
| WHO | World Health Organization |

# REFERENCES

1. TDR. First new treatment for river blindness approved by U.S. FDA in 20 years 2018 [cited 2018 June 27]. Available from: <http://www.who.int/tdr/news/2018/moxidectin-approved-as-treatment-for-river-blindness/en/>.

2. Opoku NO, Bakajika DK, Kanza EM, Howard H, Mambandu GL, Nyathirombo A, et al. Single dose moxidectin versus ivermectin for Onchocerca volvulus infection in Ghana, Liberia, and the Democratic Republic of the Congo: a randomised, controlled, double-blind phase 3 trial. Lancet. 2018. Epub 2018/01/24. doi: 10.1016/S0140-6736(17)32844-1. PubMed PMID: 29361335.

3. Ichimori K, King JD, Engels D, Yajima A, Mikhailov A, Lammie P, et al. Global programme to eliminate lymphatic filariasis: the processes underlying programme success. PLoS neglected tropical diseases. 2014;8(12):e3328. Epub 2014/12/17. doi: 10.1371/journal.pntd.0003328. PubMed PMID: 25502758; PubMed Central PMCID: PMCPmc4263400.

4. Ottesen EA, Duke BO, Karam M, Behbehani K. Strategies and tools for the control/elimination of lymphatic filariasis. Bull World Health Organ. 1997;75(6):491-503. PubMed PMID: 9509621; PubMed Central PMCID: PMCPMC2487030.

5. WHO. Global programme to eliminate lymphatic filariasis: progress report, 2017. Wkly Epidemiol Rec. 2018;93(44):589–604.

6. Ottesen EA. Lymphatic filariasis: Treatment, control and elimination. Advances in parasitology. 2006;61:395-441. Epub 2006/06/01. doi: 10.1016/s0065-308x(05)61010-x. PubMed PMID: 16735170.

7. WHO. Guideline: alternative mass drug administration regimens to eliminate lymphatic filariasis. King JD, editor: WHO/Department of control of neglected tropical diseases; 2017.

8. Thomsen EK, Sanuku N, Baea M, Satofan S, Maki E, Lombore B, et al. Efficacy, Safety, and Pharmacokinetics of Coadministered Diethylcarbamazine, Albendazole, and Ivermectin for Treatment of Bancroftian Filariasis. Clinical infectious diseases : an official publication of the Infectious Diseases Society of America. 2016;62(3):334-41. Epub 2015/10/22. doi: 10.1093/cid/civ882. PubMed PMID: 26486704.

9. King CL, Suamani J, Sanuku N, Cheng YC, Satofan S, Mancuso B, et al. A Trial of a Triple-Drug Treatment for Lymphatic Filariasis. N Engl J Med. 2018;379(19):1801-10. doi: 10.1056/NEJMoa1706854. PubMed PMID: 30403937; PubMed Central PMCID: PMCPMC6194477.

10. Edi C, Bjerum CM, Ouattara AF, Chhonker YS, Penali LK, Meite A, et al. Pharmacokinetics, safety, and efficacy of a single co-administered dose of diethylcarbamazine, albendazole and ivermectin in adults with and without Wuchereria bancrofti infection in Cote d'Ivoire. PLoS neglected tropical diseases. 2019;13(5):e0007325. doi: 10.1371/journal.pntd.0007325. PubMed PMID: 31107869.

11. Irvine MA, Stolk WA, Smith ME, Subramanian S, Singh BK, Weil GJ, et al. Effectiveness of a triple-drug regimen for global elimination of lymphatic filariasis: a modelling study. Lancet Infect Dis. 2017;17(4):451-8. doi: 10.1016/S1473-3099(16)30467-4. PubMed PMID: 28012943.

12. Kyelem D, Biswas G, Bockarie MJ, Bradley MH, El-Setouhy M, Fischer PU, et al. Determinants of success in national programs to eliminate lymphatic filariasis: a perspective identifying essential elements and research needs. Am J Trop Med Hyg. 2008;79(4):480-4. PubMed PMID: 18840733; PubMed Central PMCID: PMCPMC2694403.

13. Molyneux DH, Hopkins A, Bradley MH, Kelly-Hope LA. Multidimensional complexities of filariasis control in an era of large-scale mass drug administration programmes: a can of worms. Parasit Vectors. 2014;7:363. doi: 10.1186/1756-3305-7-363. PubMed PMID: 25128408; PubMed Central PMCID: PMCPMC4261528.

14. Horton J. Albendazole: a broad spectrum anthelminthic for treatment of individuals and populations. Curr Opin Infect Dis. 2002;15(6):599-608. doi: 10.1097/01.qco.000044779.05458.b0. PubMed PMID: 12821837.

15. Horton J, Witt C, Ottesen EA, Lazdins JK, Addiss DG, Awadzi K, et al. An analysis of the safety of the single dose, two drug regimens used in programmes to eliminate lymphatic filariasis. Parasitology. 2000;121 Suppl:S147-60. Epub 2001/06/02. PubMed PMID: 11386686.

16. Kitzman D, Cheng KJ, Fleckenstein L. HPLC assay for albendazole and metabolites in human plasma for clinical pharmacokinetic studies. J Pharm Biomed Anal. 2002;30(3):801-13. PubMed PMID: 12367706.

17. Geary TG. Ivermectin 20 years on: maturation of a wonder drug. Trends Parasitol. 2005;21(11):530-2. doi: 10.1016/j.pt.2005.08.014. PubMed PMID: 16126457.

18. Moreno Y, Nabhan JF, Solomon J, Mackenzie CD, Geary TG. Ivermectin disrupts the function of the excretory-secretory apparatus in microfilariae of Brugia malayi. Proc Natl Acad Sci U S A. 2010;107(46):20120-5. doi: 10.1073/pnas.1011983107. PubMed PMID: 21041637; PubMed Central PMCID: PMCPMC2993382.

19. Awadzi K, Edwards G, Opoku NO, Ardrey AE, Favager S, Addy ET, et al. The safety, tolerability and pharmacokinetics of levamisole alone, levamisole plus ivermectin, and levamisole plus albendazole, and their efficacy against Onchocerca volvulus. Ann Trop Med Parasitol. 2004;98(6):595-614. doi: 10.1179/000349804225021370. PubMed PMID: 15324466.

20. Kitzman D, Wei SY, Fleckenstein L. Liquid chromatographic assay of ivermectin in human plasma for application to clinical pharmacokinetic studies. J Pharm Biomed Anal. 2006;40(4):1013-20. doi: 10.1016/j.jpba.2005.08.026. PubMed PMID: 16242280.

21. Awadzi K, Edwards G, Duke BO, Opoku NO, Attah SK, Addy ET, et al. The co-administration of ivermectin and albendazole--safety, pharmacokinetics and efficacy against Onchocerca volvulus. Ann Trop Med Parasitol. 2003;97(2):165-78. doi: 10.1179/000349803235001697. PubMed PMID: 12803872.

22. Bolla S, Boinpally RR, Poondru S, Devaraj R, Jasti BR. Pharmacokinetics of diethylcarbamazine after single oral dose at two different times of day in human subjects. J Clin Pharmacol. 2002;42(3):327-31. PubMed PMID: 11865970.

23. Geary TG, Woo K, McCarthy JS, Mackenzie CD, Horton J, Prichard RK, et al. Unresolved issues in anthelmintic pharmacology for helminthiases of humans. Int J Parasitol. 2010;40(1):1-13. doi: 10.1016/j.ijpara.2009.11.001. PubMed PMID: 19932111.

24. McGarry HF, Plant LD, Taylor MJ. Diethylcarbamazine activity against Brugia malayi microfilariae is dependent on inducible nitric-oxide synthase and the cyclooxygenase pathway. Filaria J. 2005;4:4. doi: 10.1186/1475-2883-4-4. PubMed PMID: 15932636; PubMed Central PMCID: PMCPMC1173132.

25. Opoku NO, Bakajika DK, Kanza EM, Howard H, Mambandu GL, Nyathirombo A, et al. Single dose moxidectin versus ivermectin for Onchocerca volvulus infection in Ghana, Liberia, and the Democratic Republic of the Congo: a randomised, controlled, double-blind phase 3 trial. Lancet. 2018;392(10154):1207-16. doi: 10.1016/S0140-6736(17)32844-1. PubMed PMID: 29361335; PubMed Central PMCID: PMCPMC6172290.

26. Weil GJ, Bogus J, Christian M, Dubray C, Djuardi Y, Fischer PU, et al. The safety of double- and triple-drug community mass drug administration for lymphatic filariasis: A multicenter, open-label, cluster-randomized study. PLoS Med. 2019;16(6):e1002839. doi: 10.1371/journal.pmed.1002839. PubMed PMID: 31233507.

27. Budge PJ, Herbert C, Andersen BJ, Weil GJ. Adverse events following single dose treatment of lymphatic filariasis: Observations from a review of the literature. PLoS neglected tropical diseases. 2018;12(5):e0006454. doi: 10.1371/journal.pntd.0006454. PubMed PMID: 29768412; PubMed Central PMCID: PMCPMC5973625.

28. Moxidectin package Insert. Food and Drug Adminstration; 2018.

29. Hertz MI, Nana-Djeunga H, Kamgno J, Jelil Njouendou A, Chawa Chunda V, Wanji S, et al. Identification and characterization of Loa loa antigens responsible for cross-reactivity with rapid diagnostic tests for lymphatic filariasis. PLoS neglected tropical diseases. 2018;12(11):e0006963. doi: 10.1371/journal.pntd.0006963.

30. Weil GJ, Lammie PJ, Richards FO, Jr., Eberhard ML. Changes in circulating parasite antigen levels after treatment of bancroftian filariasis with diethylcarbamazine and ivermectin. J Infect Dis. 1991;164(4):814-6. Epub 1991/10/11. PubMed PMID: 1894943.

31. Chhonker YS, Sleightholm RL, Murry DJ. Bioanalytical method development and validation of moxidectin in plasma by LC-MS/MS: Application to in vitro metabolism. Biomed Chromatogr. 2019;33(2):e4389. doi: 10.1002/bmc.4389. PubMed PMID: 30238696.

**List of Appendices**

1. Prescribing Information for Study Medications
2. Informed Consent Forms
3. Safety Plan
4. DSMB Charter
5. DOLF Project Data Sharing and Publication Policy
